# Functional Divergence of Plant‐Derived *Thaumatin‐Like Protein* Genes in Two Closely Related Whitefly Species

**DOI:** 10.1002/advs.202502193

**Published:** 2025-02-28

**Authors:** Yuan Hu, Cheng Gong, Zezhong Yang, Haolin Han, Tian Tian, Xin Yang, Wen Xie, Shaoli Wang, Qingjun Wu, Xuguo Zhou, Ted C. J. Turlings, Zhaojiang Guo, Youjun Zhang

**Affiliations:** ^1^ State Key Laboratory of Vegetable Biobreeding Department of Plant Protection Institute of Vegetables and Flowers Chinese Academy of Agricultural Sciences Beijing 100081 China; ^2^ Institute of Plant Protection Tianjin Academy of Agricultural Sciences Tianjin 300381 China; ^3^ Department of Entomology School of Integrative Biology College of Liberal Arts & Sciences University of Illinois Urbana‐Champaign Urbana IL 61801‐3795 USA; ^4^ State Key Laboratory of Cotton Bio‐breeding and Integrated Utilization School of Life Sciences College of Agriculture Henan University Zhengzhou 475004 China; ^5^ Laboratory of Fundamental and Applied Research in Chemical Ecology Institute of Biology University of Neuchâtel Neuchâtel CH‐2000 Switzerland

**Keywords:** evolutionary novelty, functional divergence, horizontal gene transfer, thaumatin‐like protein, whiteflies

## Abstract

The recent discovery that various insects have acquired functional genes through horizontal gene transfer (HGT) has prompted numerous studies into this puzzling and fascinating phenomenon. So far, horizontally transferred genes are found to be functionally conserved and largely retained their ancestral functions. It evidently has not yet been considered that horizontally transferred genes may evolve and can contribute to divergence between species. Here, it is first showed that the genomes of the two widespread and agriculturally important whiteflies *Trialeurodes vaporariorum* and *Bemisia tabaci* both contain a plant‐derived *thaumatin‐like protein* (*TLP*) gene, but with highly distinct functions in these closely related pests. In *T. vaporariorum*, *TLP* has maintained a function similar to that of the plant donor, acting as an antimicrobial protein to resist fungal infection; but in sharp contrast, in *B. tabaci*, TLP has evolved into an effector that suppresses plant defense responses. These findings reveal an as‐yet undescribed scenario of cross‐species functional differentiation of horizontally transferred genes and suggest that the HGT‐mediated evolutionary novelty can contribute to ecotypic divergence and even speciation.

## Graphical abstract

Two closely related whitefly species, *Trialeurodes vaporariorum* and *Bemisia tabaci*, are found to each possess a plant‐derived *TLP* gene, but with entirely different functions. This finding of functional divergence challenges the notion that HGT genes maintain similar functions across species and shows that they can follow unique evolutionary paths that can lead to ecotypic divergence and even speciation.

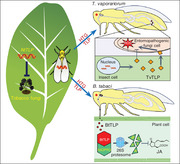

## 1 Introduction

Thanks to the advances in genetics and molecular evolution research, some of the mechanisms involved in evolutionary divergence among species, such as the emergence of new genes and the differentiation of gene functions, have been elucidated.^[^ ^1^ ^]^ A possible role of horizontal gene transfer (HGT) in these processes has been largely ignored despite the fact that HGT represents a powerful mechanism for the acquisition of new genes and can be a crucial contributor to evolutionary innovation.^[^ ^2^ ^]^ HGT not only accelerates genome evolution, enabling species to better respond to environmental pressures, but may also expand ecological niches and phenotypic plasticity.^[^ ^3^ ^]^ For example, recent evidence has revealed that horizontally transferred genes can serve in antifreeze processes in fish, the evolution of land plants, and the detoxification of plant defense compounds.^[^ ^3^, ^4^ ^]^Yet, the roles of horizontally transferred genes in functional differentiation between species remains largely unknown.

Insects are the most species‐rich group of animals on Earth, exhibiting significant ecological diversity and rapid evolutionary rates.^[^ ^5^ ^]^ They therefore can serve as ideal models for studying adaptive variations between closely related species.^[^ ^6^ ^]^ Among them are the greenhouse whitefly, *Trialeurodes vaporariorum*, and the sweet potato whitefly, *Bemisia tabaci*, two of the most widespread and agriculturally important whitefly pests in the family Aleyrodidae (Figure S1, Supporting Information).^[^ ^7^ ^]^ They cause extensive damage to various crops worldwide through phloem feeding and in particular by transmitting plant viruses and excreting honeydew.^[^ ^4^, ^8^ ^]^ The two closely related whiteflies are highly similar in morphology, with primarily only slight differences in the posture of the adult wings and the shape of the fourth instar nymphs (Figure S1, Supporting Information).^[^ ^9^ ^]^ However, they exhibit clear differences in various aspects of their biology and ecology, including host plant range, insecticide resistance, behavior, and the ability and specificity of virus transmission.^[^ ^10^ ^]^

In this study, we show that a plant‐derived horizontally transferred gene, *thaumatin‐like protein* (*TLP*), has undergone an extraordinary divergence in *T. vaporariorum* and *B. tabaci*. We found that TLP is essential for resisting pathogenic fungi in tobacco plants and serves a similar role in preventing fungal infections in *T. vaporariorum*, whereas it has undergone subfunctionalization as an effector that suppresses plant defenses in *B. tabaci*. Combined, these findings reveal a new evolutionary scenario whereby a foreign gene evolved entirely different functions and possibly contributed to ecotypic divergence between two closely related species. These new insights gained into the mechanisms of plant defense modulation and host range expansion by important pest insects also hold potential for the development of novel crop protection strategies.

## 2 Results

### 2.1 Horizontal Transfer of *TLP* from Plants to *T. Vaporariorum* and *B. Tabaci*

In line with previous studies,^[^ ^11^ ^]^ we have identified a plant‐derived gene, *TLP*, in both the genomes of *T. vaporariorum* and *B. tabaci*.^[^ ^12^ ^]^ Based on tBLASTn search, two candidate but incomplete plant‐derived *TLP* genes could also be found in a mixed population of two other whiteflies, *Pealius mori* and *Singhiella simplex*, while tBLASTn hits in other Aleyrodidae species most likely represented plant contamination (Table S2, Supporting Information). A BLASTp search against the GenBank database revealed that the closest homologs of TvTLP and BtTLP were all plant proteins. Our phylogenetic analysis confirmed that TvTLP and BtTLP cluster together with plant TLPs (Figure S2, Supporting Information). Genome analyses revealed that the *TvTLP* gene is located at scaffold 11 in the genome of *T. vaporariorum*, that it is surrounded by insect genes and that it was accurately assembled (Figure S3A, Supporting Information). Overlapping PCR amplicons of these genomic regions confirmed the assembling accuracy and ensured that *TvTLP* was indeed integrated into the *T. vaporariorum* genome (Figure S3B, Supporting Information). Furthermore, a BLASTp search against the predicted protein dataset of the *T. vaporariorum* genome showed three short exon‐like fragments similar to TvTLP (777 bp). However, the fully assembled sequence of these fragments (563 bp) could not be picked up at the cDNA level with either of the five designed full‐length primer pairs, which is indicative of a pseudogene (Table S3, Supporting Information). Moreover, we found that these fragments are tandemly arranged in a head‐to‐tail orientation with the *TvTLP* gene in the genome, which might have originated from duplication‐divergence evolution and subsequent pseudogenization.

**Figure 1:**
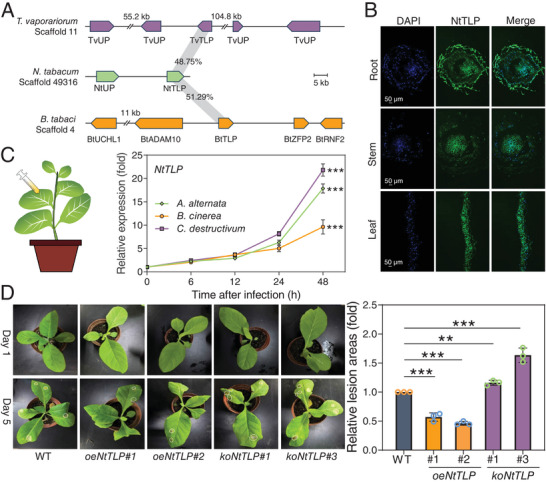
Functional analysis of the ancestral *N. tabacum TLP* gene. A) Synteny analysis of the *TLP* genes among *N. tabacum*, *T. vaporariorum* and *B. tabaci* MED. TvUP, *T. vaporariorum* uncharacterized protein; NtUP, *N. tabacum* uncharacterized protein; BtUCHL1, *B. tabaci* ubiquitin carboxyl‐terminal hydrolase L1; BtADAM10; *B. tabaci* disintegrin and metalloproteinase domain‐containing protein 10; BtZFP2, *B. tabaci* zinc finger protein 2; BtRNF2, *B. tabaci* E3 ubiquitin‐protein ligase RING2. B) Immunofluorescence localization of NtTLP in the root, stem and leaf of *N. tabacum*. Nuclei are stained with DAPI (blue), green is the positive signal for anti‐NtTLP. C) The transcript level of *NtTLP* after infection by *A. alternata*, *B. cinerea* and *C. destructivum*. The significance is a comparison of the transcript levels of *NtTLP* at 0 and 48 h. D) Virulence assay of *Colletotrichum destructivum* on leaves of *N. tabacum* K326 (WT), two overexpressing *NtTLP* (*oeNtTLP#1*, *oeNtTLP#2*) and two *NtTLP* CRISPR/Cas9 mutants (*koNtTLP#1*, *koNtTLP#3*) tobacco plants. The photographs were taken on day 1 and day 5, and the disease assay was evaluated by lesion area. The white dotted box shows the location of the spot. Date are means ± SEM, *n* = 6 (c) biologically independent samples, **P < 0.01, ***P < 0.001, one‐way ANOVA with Tukey's test was used for comparison.

The *BtTLP* was accurately assembled and highly consistent across different *B. tabaci* cryptic species. Furthermore, overlapping PCR amplicons of these genomic regions confirmed the assembling accuracy and ensured that *BtTLP* were indeed integrated into the *B. tabaci* MED genome (Figures S3C and S4, Supporting Information). Genomic regions of the *BtTLP* gene and surrounding genes of *B. tabaci* MED share highly conserved synteny with *B. tabaci* MEAM1 (Figure S3D, Supporting Information), confirming that *TLP* was acquired before the divergence of the *B. tabaci* cryptic species.^[^ ^11^ ^]^ Phylogenetic analyses in previous studies could not unequivocally determine whether the *TLP* gene originated from two distinct HGT events or was acquired before the split of *T. vaporariorum* and *B. tabaci* ≈86 million years ago.^[^ ^11^, ^13^ ^]^ (Figure S3E, Supporting Information). Also, the presence of different surrounding coding genes and non‐coding sequences suggests a lack of synteny between the *TvTLP* and *BtTLP* gene regions (**Figure** **1**A). To conclude, we show that *TvTLP* and *BtTLP* genes are not plant gene contaminants, but *T. vaporariorum* and *B. tabaci* have horizontally acquired a *TLP* gene from host plants, either independently or derived from a common ancestor.

### 2.2 *Nicotiana Tabacum* TLP is an Antifungal Protein

To systematically investigate the function of the *TLP* gene, we used *Nicotiana tabacum* as a model system given its significance as a major host for whiteflies. We first screened for and obtained a tobacco *TLP* gene with the highest homology to *BtTLP* and *TvTLP*, which we named *NtTLP* (GenBank accession no. PP778349). It is noteworthy that NtTLP and TvTLP have an amino acid similarity of 48.75%, and NtTLP and BtTLP have an amino acid similarity of 51.29% (Figure 1A). The full‐length cDNA sequences of the tobacco *TLP* gene (990 bp) was successfully cloned, and sequence analysis revealed that this TLP protein contain conserved thaumatin domain and 16 cysteine residues (Figure S5A–C, Supporting Information). Immunofluorescence analysis demonstrated that NtTLP was located in root, stem and leaves of *N. tabacum* (Figure 1B). Further, NtTLP had the best BLASTp hit (E‐value of E‐136) with the pathogenesis‐related thaumatin‐like protein *Thau3* (AT1G75800) in the protein database of *A. thaliana* (TAIR, accessed July 12, 2024), with *Thau1/2/3/4 mutant* showing increased susceptibility to *Pseudomonas syringae*.^[^ ^14^ ^]^ Previous studies have also concluded that antimicrobial activity might be the major function of plant *TLPs*.^[^ ^15^ ^]^ To assess the antifungal activity of *NtTLP*, we analyzed transcript levels of *NtTLP* upon pathogen infestation and found that these were significantly induced in tobacco upon infection by three representative tobacco fungi, namely *Alternaria alternata*, *Botrytis cinerea* and *Colletotrichum destructivum* (Figure 1C). To further confirm the role of *NtTLP* in resistance to fungal pathogens, two tobacco lines overexpressing *NtTLP* (*oeNtTLP#1*, *oeNtTLP#2*) and two CRISPR/Cas9 mutants of *NtTLP* (*koNtTLP#1*, *koNtTLP#3*) were generated in the *N. tabacum* K326 (WT) background (Figure S6, Supporting Information). The lesion areas caused by fungal infection in the *NtTLP*‐overexpressed transgenic tobacco lines were significantly smaller compared to the WT at 5 days post‐infection. Conversely, CRISPR/Cas9‐generated *NtTLP* mutant lines were exhibited significantly greater susceptibility to infection compared to WT (Figure 1D). These results indicated that *NtTLP* significantly contributes to *N. tabacum* resistance against fungal pathogens.

### 2.3 The Expression Patterns of TLP are Different in *T. Vaporariorum* an*d B. Tabaci*

**Figure 2:**
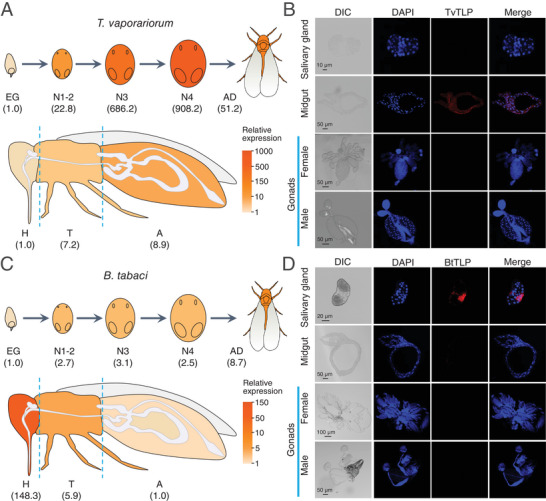
The expression patterns of TvTLP in *T. vaporariorum* and BtTLP in *B. tabaci*. A) The transcript levels of *TvTLP* in eggs (EG), 1^st^‐ and 2^nd^‐instar nymphs (N1‐2), 3^rd^‐instar nymphs (N3), 4^th^‐instar nymphs (N4), adults (AD) of *T. vaporariorum* and head (H), thorax (T) and abdomen (A) of *T. vaporariorum* adults were determined by qPCR. The relative expression level (fold change) was calculated based on the lowest measured expression value (Developmental stages: EG; Tissues: H), which was arbitrarily assigned a value of 1. The numbers in brackets represent the mean expression values. B) Immunofluorescence localization of TvTLP protein in salivary gland, midgut and gonads of *T. vaporariorum*. Nuclei are shown in blue, red is the positive signal for anti‐TvTLP. C) The transcript levels of *BtTLP* in eggs (EG), 1^st^‐ and 2^nd^‐instar nymphs (N1‐2), 3^rd^‐instar nymphs (N3), 4^th^‐instar nymphs (N4), adults (AD) of *B. tabaci* and head, thorax and abdomen of *B. tabaci* adults were determined by qPCR. The relative expression level (fold change) was calculated based on the lowest measured expression value (Developmental stages: EG; Tissues: A), which was arbitrarily assigned a value of 1. The numbers in brackets represent the mean expression values. D) Immunofluorescence localization of BtTLP protein in salivary gland, midgut, and gonads of *B. tabaci*. Nuclei are shown in blue, red is the positive signal for anti‐BtTLP.

The full‐length cDNA sequences of the *TvTLP* gene (777 bp, GenBank accession no. PP778350) and *BtTLP* gene (726 bp, GenBank accession no. PP778351) were successfully cloned, and sequence analysis revealed that both TLP proteins contain conserved thaumatin domain and 16 cysteine residues, whereas the SignalP analysis showed that the signal peptides were different in signal peptide length (39 aa vs 21 aa) as well as amino acid similarity (10.26%) between the two whitefly species (Figure S5, Supporting Information). To investigate the functional role of the *T. vaporariorum TLP* and *B. tabaci TLP*, the expression profiles of *TvTLP* and *BtTLP* were examined. qPCR results showed that *TvTLP* was most highly expressed in the 4^th^ instar nymph and the abdomen of the adult whitefly (**Figure** **2**A; Figure S7A,B, Supporting Information). Immunofluorescence showed that the TvTLP protein was specifically located in the midgut, which is a major site for the immunologic process,^[^ ^16^ ^]^ but not in the salivary gland and gonads (Figure 2B). In contrast, qPCR analysis showed that *BtTLP* is highly expressed in the head of adult *B. tabaci* (Figure 2C; Figure S7C,D, Supporting Information), and immunofluorescence showed that BtTLP protein is specifically located in the salivary gland but not in the midgut and gonads (Figure 2D), suggesting it mainly plays a role in the salivary gland.

### 2.4 TvTLP is an Antifungal Protein that Enables *T. Vaporariorum* to Resist Entomopathogenic Fungi

**Figure 3:**
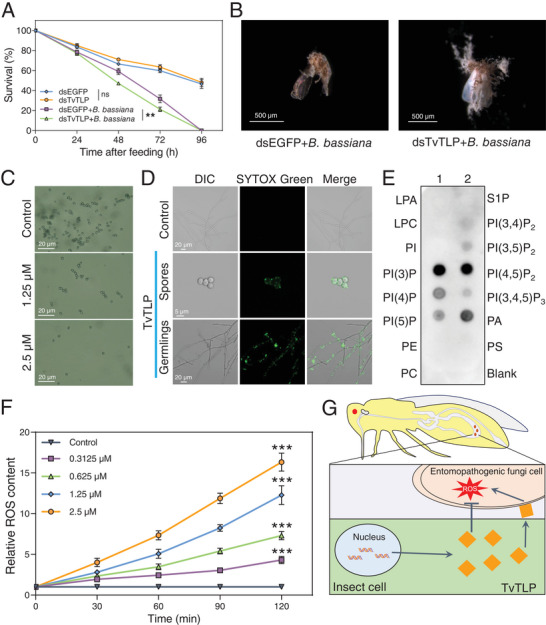
The fungicidal effects of TvTLP against *B. bassiana* via the induction of cell permeabilization and ROS production. A) Survival rate of *T. vaporariorum* that were fed either dsEGFP or dsTvTLP, with or without *B. bassiana*. B) *B. bassiana*‐covered cadavers of *T. vaporariorum* adults after being treated with dsEGFP and dsTvTLP. C) Representative microscopic images showing the inhibition of *B. bassiana* growth 48 h after treatment with 1.25 and 2.5 µm of TvTLP, and no TvTLP protein treatment as control. D) Confocal microscopy images of SYTOX Green (SG) uptake in *B. bassiana* conidia and germlings treated with 2.5 µm TvTLP, no TvTLP protein treatment as control. SG (green) labeled nuclei in conidia and germlings indicate TvTLP plasma membrane permeability. E) PIP Strip assay of TvTLP protein binding to membrane lipids. The intensity of the black dots correlates with the strength of the binding ability. LPA, lysophosphatidic acid; LPC, lysophosphatidylcholine; PI, phosphatidylinositol; PI(3)P, phosphatidylinositol 3‐phosphate; PI(4)P, phosphatidylinositol 4‐phosphate; PI(5)P, phosphatidylinositol 5‐phosphate; PE, Phosphatidylethanolamine; PC, Phosphatidylcholine; S1P, sphingosine 1‐phosphate; PI(3,4)P2, phosphatidylinositol 3,4‐bisphosphate; PI(3,5)P2, phosphatidylinositol 3,5‐bisphosphate; PA, Phosphatidic acid; PS, Phosphatidylserine; PI(4,5)P2, phosphatidylinositol 4,5‐bisphosphate; PI(3,4,5)P3, phosphatidylinositol 3,4,5‐trisphosph; PA, phosphatidic acid; PS, phosphatidylserine. F) ROS content in *B. bassiana* conidia treated with different concentrations of TvTLP. G) A proposed model for *T. vaporariorum* employing TvTLP to defend itself against entomopathogenic fungi. Upon infection with the *B. bassiana* pathogen, *TvTLP* binds to the plasma membrane, inducing membrane permeabilization and ROS production, thus decreasing fungal pathogenicity. Date are means ± SEM, *n* = 6 (A) biologically independent samples, ns, not significant, **P < 0.01, ***P < 0.001, one‐way ANOVA with Tukey's test was used for comparison.

The different expression patterns of TvTLP in *T. vaporariorum* and BtTLP in *B. tabaci* prompted us to examine possible differences in function. To test for the role of *TvTLP* in host defense against fungal infection, as is the case for *NtTLP* in *N. tabacum*, we infected *T. vaporariorum* with two typical entomopathogens *Beauveria bassiana* and *Metarhizium robertsii* and found that this significantly increased the transcript levels of *TvTLP* after 48 h (Figure S8A,B, Supporting Information). We then performed RNA interference (RNAi) experiments and qPCR analysis showed that the transcript levels of *TvTLP* were significantly reduced after RNAi for 72 h (Figure S8C, Supporting Information). Then, we monitored survival of *T. vaporariorum* that were fed either dsEGFP or dsTvTLP, with or without the conidia of *B. bassiana* and *M. robertsii*. Under aseptic conditions, silencing of *TvTLP* had no effect on the survival rate of *T. vaporariorum* compared with controls. However, when *T. vaporariorum* were treated with *B. bassiana* and *M. robertsii*, silencing caused a significant decrease in survival rate (**Figure** **3**A; Figure S8D, Supporting Information). Among the infected dead adults, the proportion of pathogen‐covered cadavers in the dsEGFP group was significantly less than that in the dsTvTLP group (Figure 3B; Figure S8E, Supporting Information). We also constructed transgenic plants to silence *TvTLP* causing a significantly decrease in the transcript level of *TvTLP* after *T. vaporariorum* had fed on transgenic‐*TvTLP* tobacco plants (Figure S9A–C, Supporting Information). In agreement with this, knockdown of *TvTLP* by transgenic plants considerably decreased the survival rate and hatching success of *T. vaporariorum* when exposed to *B. bassiana* and *M. robertsii* (Figure S9D–G, Supporting Information). As for the adults, among the dead *T. vaporariorum* nymphs, the proportion of pathogen‐covered cadavers in the dsEGFP group was significantly less than that in the dsTvTLP group (Figure S9H,I, Supporting Information). These results imply that *TvTLP* is important for *T. vaporariorum* to defend itself against fungi.

To further confirm the role of *TvTLP* in antifungal activity, the growth of *B. bassiana* and *M. robertsii* were inhibited by ectopic expressed recombinant TvTLP protein, showing half‐maximal inhibitory concentration (IC_50_) values of 1.531 and 2.043 µm, respectively (Figure S10A, Supporting Information). Furthermore, the resazurin cell viability assay revealed that *B. bassiana* conidia lose their cellular metabolic activity at a concentration of 2.5 µm TvTLP (Figure 3C; Figure S10B, Supporting Information). The SYTOX Green (SG) nucleic acid stain, which penetrates cells with compromised plasma membranes, was used to evaluate the ability of TvTLP to penetrate fungal cell membranes. The overall results from staining conidia and germlings with SG (Figure 3D) revealed that TvTLP protects against fungi by permeabilizing its membranes.

Previously reported plant TLPs display β‐1,3‐glucanase activity.^[^ ^15b^ ^]^ We observed that NtTLP and TvTLP also possess β‐1,3‐glucanase activity and that this activity increases with protein concentration (Figure S10C,D, Supporting Information). It is known that antifungal peptides can bind to membrane phospholipids and induce the production of reactive oxygen species (ROS).^[^ ^17^ ^]^ To assess whether TvTLP also has the ability to bind to membrane phospholipids, a protein‐lipid overlay assay was conducted and revealed that TvTLP strongly binds to PI(3)P, PI(4,5)P2 and phosphatidic acid (PA). It also weakly bound to PI(4)P, PI(3,4,5)P3, PI(5)P, PI(3,4)P2, and PI(3,5)P2 (Figure 3E; Figure S10E, Supporting Information). Moreover, rapid induction of ROS was observed within 30 min, which increased in both a time‐dependent and dose‐dependent manner (Figure 3F), demonstrating the elicitation of ROS in conidia following TvTLP treatment. These observations show that TvTLP functions as an antifungal protein that targets one or more membrane phospholipids, disrupts the plasma membrane, and induces ROS production as a defense against fungi (Figure 3G).

### 2.5 BtTLP is a Salivary Protein that Suppresses Plant Defense Responses and Enhances the Performance of *B. Tabaci* on its Host Plants

**Figure 4:**
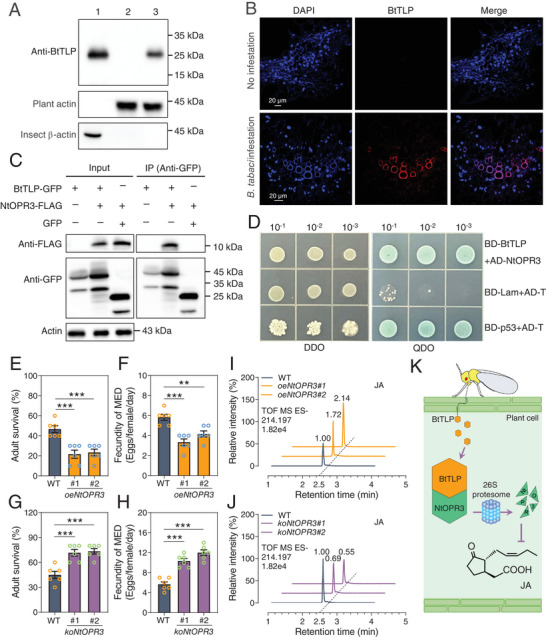
The *B. tabaci* MED effector BtTLP interacts with NtOPR3 to inhibit JA synthesis. A) Western blot analysis of BtTLP secretion. Lane 1, protein extracted from *B. tabaci* adults; lane 2, protein extracted from uninfested tobacco leaves; lane 3, protein extracted from *B. tabaci*‐infested tobacco leaves. B) Immunofluorescence localization of BtTLP in *B. tabaci* MED‐infested and uninfested tobacco leaves. DAPI is shown in blue, red is the positive signal for anti‐BtTLP. C) Co‐IP assays showing the interaction between BtTLP and NtOPR3 in *N. benthamiana* leaves. D) Y2H assays showing the interaction between BtTLP and NtOPR3. The bait plasmid pGBKT7 (BD) carrying BtTLP and the prey plasmid pGADT7 (AD) carrying NtOPR3 were co‐transformed into the yeast strain Y2H Gold. p53 and SV40 large T antigen co‐transformed yeast cells as positive controls, lamin and SV40 large T antigen co‐transformed yeast cells as a negative control. DDO, SD/−Leu/‐Trp; QDO, SD/‐Trp/‐Leu/‐His/‐Ade. E,F) Survival rate (E) and fecundity (F) of *B. tabaci* MED on WT and two *NtOPR3*‐overexpressed (*oeNtOPR3#1*, *oeNtOPR3#2*) transgenic tobacco plants after 7 days. G,H) The survival (G) and fecundity (H) of *B. tabaci* MED on WT and two *NtOPR3* knockout (*koNtOPR3#1*, *koNtOPR3#2*) transgenic tobacco plants after 7 days. I,J) The content of JA in WT and two *NtOPR3*‐overexpressed (*oeNtOPR3#1*, *oeNtOPR3#2*) (I) and two knockout (*koNtOPR3#1*, *koNtOPR3#2*) (J) transgenic tobacco plants. The numbers above each peak indicates the relative JA level calculated by the ratio of peak areas. K) *B. tabaci* releases TLP into the plant cell and interacts with NtOPR3, BtTLP causes the degradation of NtOPR3 by 26S proteasome, thereby inhibiting JA signaling pathway. Date are means ± SEM, *n* = 6 (E‐H) biologically independent samples, *P < 0.05, **P < 0.01, one‐way ANOVA with Tukey's test was used for comparison.

We also tested the possible role of *BtTLP* in host defense against fungal infection and compared it to *TvTLP* in *T. vaporariorum*. In contrast to the important role of *TvTLP* in *T. vaporariorum* immunity, we found that *BtTLP* is associated with feeding in *B. tabaci*, as the transcript level of *BtTLP* was significantly increased after 2 h feeding on tobacco (Figure S11, Supporting Information). This suggested that *BtTLP* may be a salivary protein. To test this, Western blot was used to determine whether BtTLP is secreted into plants. The BtTLP protein was detected in *B. tabaci* bodies and in *B. tabaci‐*infested tobacco leaves but was not found in uninfested tobacco leaves (**Figure** **4**A). Immunofluorescence experiment confirmed the presence of the BtTLP protein in *B. tabaci‐*infested but not in uninfested tobacco leaf cells (Figure 4B).

To determine whether the salivary protein BtTLP has an effect on plant defense responses, we performed agrobacterium‐mediated transient expression of BtTLP in *N. benthamiana* (Figure S12A, Supporting Information). The results showed that BtTLP inhibits the hypersensitive response induced by the immunity elicitor Bax. A subcellular localization study confirmed that BtTLP is located in the nucleus and cytoplasm of *N. benthamiana* cells (Figure S12B, Supporting Information). These results imply that BtTLP is an effector that may interact with proteins in the plant cytoplasm and nucleus.

To investigate whether *BtTLP* indeed enhances *B. tabaci* performance on host plants, specific dsRNA targeting *BtTLP* was directly fed to *B. tabaci* adults. This dramatically decreased mRNA expression levels of the *BtTLP* after 48 h of feeding as compared to adults fed with dsEGFP (Figure S13A, Supporting Information). Importantly, *B. tabaci* adults fed with dsBtTLP had a significant reduced survival rate on tobacco plants from day 3 to day 7 compared to those that were feeding on artificial diet (Figure S13B,C, Supporting Information). Electrical penetration graphs (EPG) to monitor the feeding behavior of *B. tabaci* adults on tobacco showed that the phloem feeding waveform was interrupted in *B. tabaci* that were fed with dsBtTLP (Figure S13D,E, Supporting Information), whereas there was no difference in non‐phloem phase parameters between dsEGFP and dsBtTLP fed *B. tabaci* (Figure S13F, Supporting Information). Specifically, the phloem phase E2 duration and the total E duration were reduced significantly in dsBtTLP fed *B. tabaci* (Figure S13G, Supporting Information), which implies that BtTLP increases phloem‐feeding in *B. tabaci*.

Two *BtTLP*‐overexpressed transgenic tobacco lines (*oeBtTLP#3*, *oeBtTLP#5*) and two *BtTLP*‐silenced transgenic lines (dsBtTLP#3, dsBtTLP#10) were chosen for further analyses (Figure S14, Supporting Information). The survival and fecundity of *B. tabaci* fed on *BtTLP*‐overexpressed tobacco lines were significantly higher than those on WT (Figure S15A,B, Supporting Information). On the contrary, survival and fecundity of *B. tabaci* fed on *BtTLP*‐silenced transgenic lines were significantly lower than on dsEGFP transgenic tobacco (Figure S15C,D, Supporting Information). These results show that *BtTLP* enhances host plant suitability for *B. tabaci* by regulating plant defense responses.

### 2.6 BtTLP Suppresses Plant Defense Responses by Manipulating Defense Hormone Production

Specific experiments were conducted to determine whether *BtTLP* regulates plant defense responses by manipulating the salicylic acid (SA) and jasmonic acid (JA) signaling pathways, which are the main pathways involved in defenses against phloem‐feeding insects.^[^ ^18^ ^]^ We examined the content of SA, JA and jasmonoyl‐L‐isoleucine (JA‐Ile) in *BtTLP* overexpression transgenic plants and tobacco plants fed by *BtTLP‐*silenced *B. tabaci*. Overexpression of *BtTLP* in tobacco plants significantly decreased the JA and JA‐Ile content while it increased the SA content compared to the WT (Figures S15E and S16A,B, Supporting Information). In addition, we found that JA‐related marker genes were downregulated while SA‐related marker genes were upregulated (Figure S16C–H, Supporting Information). Conversely, the *BtTLP‐*silenced *B. tabaci* were fed on tobacco plants, the plants’ JA and JA‐Ile content increased while SA content decreased (Figure S15F and S16I,J, Supporting Information), and the JA‐related marker genes were upregulated while SA‐related marker genes were downregulated (Figure S16K–P, Supporting Information). Thus, these results reveal that *BtTLP* enhances *B. tabaci* host adaptation by regulating plant defense through the fine‐tuning of both the SA and JA signaling pathways (Figure S15G, Supporting Information).

To understand how BtTLP manipulates plant defense, we used the yeast two‐hybrid (Y2H) technique combined with immunoprecipitation mass spectrometry (IP‐MS) to screen for potential target proteins in tobacco (Figure S17A–C, Supporting Information). We identified a large number of potential target proteins, but only two proteins present in both BtTLP IP‐MS and Y2H (Figure S17D and Table S4, Supporting Information), interestingly, including a *N. tabacum* 12‐oxophytodienoate reductase (OPR) and a *N. tabacum* heat shock protein (HSP). The OPR3 has previously been shown to be involved in JA synthesis.^[^ ^19^ ^]^ We therefore focused on the OPR. Gene cloning and evolutionary tree analysis confirmed that this gene belongs to the OPR family and is closely related to the OPR3 of subgroup I (Figure S18, Supporting Information), henceforth called NtOPR3 (GenBank accession no. PP778352). To confirm the direct interaction between BtTLP and NtOPR3, point‐to‐point Y2H, bimolecular fluorescence complementation (BiFC), co‐immunoprecipitation (Co‐IP) and luciferase complementation (LUC) assays were performed, which demonstrated specific binding of BtTLP to NtOPR3 (Figure 4C,D; Figure S19, Supporting Information).

We obtain further insights into the effect of BtTLP on NtOPR3 function, by co‐expressing NtOPR3‐mcherry and BtTLP‐GFP in *N. benthamiana*. Confocal microscopy analyses revealed that NtOPR3‐mCherry is mainly located in the cytoplasm and peroxisome (Figure S20A, Supporting Information). Such analyses also showed that when BtTLP‐GFP and NtOPR3‐mCherry were co‐expressed in *N. benthamiana*, the signal of NtOPR3‐mCherry was attenuated compared to the GFP control (Figure S20A, Supporting Information). In addition, NtOPR3‐Flag was co‐expressed with BtTLP‐GFP in *N. benthamiana*, as Western blot analysis showed that a lower amount of NtOPR3 accumulated in the presence of BtTLP‐GFP than in the GFP control (Figure S20B, Supporting Information). These results indicated that NtOPR3 degradation occurs in a BtTLP‐dependent manner.

The ubiquitin‐proteasome system and autophagy are the primary pathways for protein degradation in plant cells and the ubiquitin‐proteasome system serves as a crucial post‐translational mechanism governing plant immune responses.^[^ ^20^ ^]^ The 26S proteasome inhibitor MG132 was used to test if this is also the case in the observed plant defense suppression. We found that the protein level of NtOPR3 can be restored when co‐infiltrated with BtTLP‐GFP upon treatment with the proteasome inhibitor MG132 (Figure S20C, Supporting Information). These results strongly suggest that BtTLP causes degradation of NtOPR3 via the 26S proteasome, thereby inhibiting the JA signaling pathway.

Our discovery that NtOPR3 as a potential target of BtTLP prompted us to further investigate its biological role in plant defense. Two tobacco lines overexpressing *NtOPR3* (*oeNtOPR3#1*, *oeNtOPR3#2*) and two CRISPR/Cas9 mutants of *NtOPR3* (*koNtOPR3#1*, *koNtOPR3#2*) were generated in the *N. tabacum* K326 (WT) background (Figure S21, Supporting Information). The survival rates and fecundity of *B. tabaci* on *NtOPR3*‐overexpressed plants were considerably lower than those on WT (Figure 4E,F). Conversely, the survival and fecundity of *B. tabaci* were increased significantly on *NtOPR3* knockout plants (Figure 4G,H). This was in agreement with the fact that contents of JA and JA‐Ile were increased in *NtOPR3*‐overexpressed lines (Figure 4I; Figure S22A, Supporting Information), whereas they were reduced in the *NtOPR3* knockout lines (Figure 4J; Figure S22B, Supporting Information). However, there were no differences among SA levels in *NtOPR3*‐overexpressed or knockout plants (Figure S22C,D, Supporting Information). In addition, the JA‐related marker genes were upregulated in *NtOPR3*‐overexpressed lines and downregulated in the *NtOPR3* knockout lines, whereas SA‐related marker genes did not change in *NtOPR3* overexpression and knockout plants (Figure S23, Supporting Information). Collectively, the presented results show that *B. tabaci* releases the effector of BtTLP into the plant cell, which causes degradation of NtOPR3 via the 26S proteasome, and the degradation of NtOPR3 affect JA synthesis and the JA signaling pathway (Figure 4K).

## 3 Discussion

**Figure 5:**
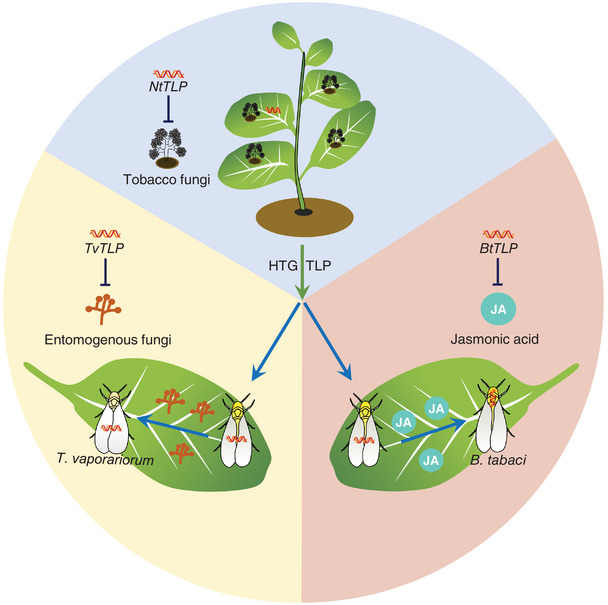
A model of how plant‐derived *TLP* genes functionally diverged between *T. vaporariorum* and *B. tabaci*. *TLP* serves antifungal functions in *N. tabacum*. Both *T. vaporariorum* and *B. tabaci* acquired a plant‐derived *TLP* gene through horizontal gene transfer. Originally, *T. vaporariorum* and *B. tabaci* adapt in parallel to similar environments. Over time, *T. vaporariorum* retains the antifungal function of the *TLP* gene, while the *TLP* gene in the closely related species *B. tabaci* undergoes subfunctionalization and assumes the role as an effector to suppress the JA signaling pathway in host plants.

In this study, we functionally characterized the horizontal transfer gene *TLP* in two closely related whitefly species *B. tabaci* and *T. vaporariorum* (**Figure** **5**). In plants, TLP belongs to the plant pathogenesis‐related protein (PR) superfamily and is a highly conserved immunity‐related protein with antifungal activity.^[^ ^15^ ^]^ Here, we first report a similar antifungal function of a plant‐derived TLP in the whitefly *T. vaporariorum*, consistent with its homolog in tobacco. The molecular mechanism underlying TvTLP's antifungal effect in the *T. vaporariorum* is shown to be the disruption the pathogen membranes, leading to an increase in ROS levels within the pathogen, ultimately causing the fungicidal activity. The plant‐derived *TLP* gene in *T. vaporariorum* is thus a textbook example of how HGT can contribute to the adaptive evolution of the receiver, offering novel immunity tools. Whether TvTLP has retained its ancestral antifungal activity against plant pathogens remains to be further investigated. If so, unveiling the pivotal role of TvTLP in fungal immunity provides valuable insights that could possibly be exploited to enhance crop resistance against fungal pathogens.

By contrast, for *B. tabaci*, we found that TLP functions as an effector that suppresses the plant JA signaling pathway while stimulating the plant SA signaling pathway. Similar to other insect effectors, it enables them to enhance host plant suitability and most likely has contributed to the vast expansion of *B. tabaci*’s host plant range.^[^ ^7^, ^21^ ^]^ The molecular mechanisms of *B. tabaci* effector‐mediated suppression of the plant defense pathway are complex.^[^ ^8^, ^22^ ^]^ The majority of characterized whitefly effectors activate SA signaling or suppress JA‐defenses to promote whitefly success.^[^ ^8^, ^23^ ^]^ Here, we show that BtTLP directly binds to the key JA synthesis enzyme NtOPR3 and thus affects JA production in tobacco. Hence, we reveal a novel mechanism by which *B. tabaci* can modulate plant defense responses by manipulating JA synthesis. It should be noted that a very recent study has shown that BtTLP, similar to TvTLP, also has glucanase activity,^[^ ^11^ ^]^ and we therefore cannot fully exclude that BtTLP has a dual function, exhibiting antifungal activity as well as modulating plant defenses. The discovery that BtTLP plays a crucial role in suppressing JA‐related plant immune responses highlights its potential as a molecular target for pest management strategies. For instance, blocking BtTLP activity should reduce *B. tabaci*’s ability to suppress plant defenses, thereby increasing plant resistance to whitefly infestations.

We can think of three non‐exclusive reasons why the *TLP* genes have evolved different functions in *T. vaporariorum* and *B. tabaci*. First, the two whiteflies may have differently adapted to environmental forces that they are typically confronted with.^[^ ^24^ ^]^ *T. vaporariorum* may be more prone to encounter entomopathogenic fungi in its habitats and therefore greatly benefits from an antifungal function. *B. tabaci*, on the other hand, may benefit much more from TLP as an effector to suppress the defenses of its exceptionally wide range of more than 600 host plant species. Second, differences in gene expression regulation may be key to the functional divergence of the *TLP* genes. Although both *TvTLP* and *BtTLP* genes were acquired from plants, they only share 43% amino acid similarity and differ significantly the signal peptide regions and even in their upstream or downstream regulatory sequences.^[^ ^12^ ^]^ These sequence dissimilarities are likely to result in significant differences in expression patterns of the *TLP* genes in different tissues and developmental stage. Finally, the *TLP* genes may differ in their interaction with various molecular chaperones or signaling pathways to respond to distinct stresses experienced by the whiteflies.^[^ ^8^, ^25^ ^]^ In sum, the functional divergence of *TLP* genes in *T. vaporariorum* and *B. tabaci* is likely to be the result of the combined effects of multiple factors during their long‐standing adaptive evolution. The disclosed divergence implies an important evolutionary role of horizontal gene transfer and illustrates that HGT genes may acquire totally new functions. It is likely that further investigations into HGT genes will reveal similar evolutionary scenarios that could shed new light on how functional divergence has contributed to key difference among closely related species.

The distribution of *TLP* genes in insects is somewhat ambiguous, as it is only present in certain insects from four insect orders.^[^ ^15b^ ^]^ The inherent functional diversification of *TLP* genes in plant ancestors complicates the tracing of their origins, as HGT genes in plants may already exhibit diverse functions, making it challenging to determine their source. Indeed, previous studies were also inconclusive whether the *TLP* gene was derived from a common ancestor or independently acquired from plants by both whitefly species.^[^ ^11^, ^13^ ^]^ The first option involves *TLP* acquisition by the common ancestor of *T. vaporariorum* and *B. tabaci*, followed by *TLP* loss in several whitefly lineages. This seems unlikely because we show that the plant‐derived *TLP* gene is beneficial to both whitefly species and should be to other whitefly species too, as they all encounter entomopathogenic fungi and are herbivorous. The alternative, independent acquisitions of a plant *TLP*, seems more plausible. A recent study reported that another insect species, *Thrips palmi* (Thysanoptera) also contains a plant *TLP* gene in its genome,^[^ ^11^ ^]^ illustrating independent *TLP* acquisitions are not uncommon. We suspect the same for *BtTLP* and *TvTLP* because of the lack of synteny, as well as difference in their signal peptides, *cis*‐regulatory sequences, expression patterns, and functional roles. Analyzing the regulatory differences between both *TLP* genes may provide new clues as to whether they were acquired independently or originated from a common ancestor. Moreover, given that the Cretaceous period witnessed the rise and rapid diversification of flowering plants (angiosperms),^[^ ^26^ ^]^ this speciation boom likely created new ecological niches and fostered novel interactions between insects and plants, which may have facilitated HGT events between species. Changes in environmental conditions, including elevated atmospheric CO₂ levels and higher temperatures,^[^ ^27^ ^]^ may have driven adaptations in plants and insects, in part facilitated by gene exchange. It would be interesting to study fossil records of ancient plant lineages from this era to possibly shed new light on the origins of *TLP* genes and the factors driving HGT. Currently, the specific plant source and timing of the HGT event remain unclear and as yet there is no reliable method to determine whether the *TLP* gene was acquired independently by the two whiteflies or derived from a common ancestor. Yet, it seems evident that the difference in function of the *TLP* gene in the two closely related whiteflies has important implications for their ecological niche.^[^ ^28^ ^]^ Our findings also highlight that, while the functions of HGT genes within a single species have been well‐documented, there is a need for cross‐species HGT research to investigate the diverse roles of HGT genes may play in different species, in particular in the divergence between species.

Most insights into HGTs have emerged from large‐scale genome analyses conducted over recent years.^[^ ^12^, ^29^ ^]^ These studies have provided a wealth of information on the genetic exchanges that have occurred, not only within but also across different domains of life. While putative functions of HGT, such as antibiotic resistance, immunity, and environmental adaptability, have been documented in both prokaryotic and eukaryotic species,^[^ ^30^ ^]^ most studies have focused on single recipient species and the retention of ancestral functions. Whether horizontally transferred genes can evolve and undergo significant functional changes within recipient species has remained unexplored. Our study appears to be a first to show that foreign genes can acquire distinctly different roles across different species. It challenges the notion that conserved HGT genes maintain similar functions across species and highlights that HGT genes may follow unique evolutionary trajectories. The differential expression patterns of a foreign gene in closely related whitefly species can also lead to the key differences in adaptive traits such as those that affect immunity and host range, which may constitute important drivers underlying ecological niche differentiation.^[^ ^10c^ ^]^ Many aspects of distinct evolutionary trajectories followed by closely related species remain unexplored. Future research necessitates additional examples to solidify the notion that HGT events can contribute to the molecular foundations underlying species divergence.

## 4 Experimental Section

### Plants, Insects, and Fungal Culture

Tobacco seeds (*Nicotiana tabacum* K326 and *Nicotiana benthamiana*) were obtained from the laboratory collection. Each plant was cultivated individually in pots under natural lighting supplemented with artificial illumination.

The strain of *Trialeurodes vaporariorum* had been maintained on *N. tabacum* K326 in the laboratory since 2018. It was initially provided by Yifan Zhai (Institute of Plant Protection, Shandong Academy of Agricultural Sciences, China). The strain of *Bemisia tabaci* MED was originally collected from Beijing, China, in 2009, and then it was reared on *N. tabacum* K326 to establish a tobacco strain. All of the *B. tabaci* and *T. vaporariorum* strains were reared in a controllable glasshouse at 26 ± 1 °C, a photoperiod of L16: D8 and 60% ± 10% relative humidity (RH).

The fungal strains *Beauveria bassiana* ARSEF 2860 and *Metarhizium robertsii* ARSEF 2575 were maintained on potato dextrose agar (PDA) at 25 ± 1 °C. The fungal strains *Alternaria alternata SX1‐8*, *Botrytis cinerea*, and *Colletotrichum destructivum* were maintained on PDA at 28 ± 1 °C. Fungal conidia were harvested by flooding the fungal growth plates with 0.01% Tween‐80 sterilized water. The spore suspension of each fungal pathogen was adjusted to equivalent spore density using a hemocytometer.

### RNA Extraction and cDNA Synthesis

Total RNA was extracted by TRIzol Reagent (Invitrogen) according to the manufacturer's protocol. The quality of the isolated RNA samples was assessed with a NanoDrop 2000c spectrophotometer (Thermo Fisher Scientific) and by 1.5% agarose gel electrophoresis. First strand cDNA was synthesized with the PrimeScript II 1st strand cDNA Synthesis Kit (TaKaRa) following the manufacturer's protocol for gene cloning and qPCR detection. Then, the synthesized cDNA was stored at −20 °C until used.

### Gene Identification and Cloning

The *NtTLP* gene was found in the Solanaceae genomics network (https://solgenomics.net/). The *TvTLP* and *BtTLP* genes were found in the previously sequenced *T. vaporariorum* genome and *B. tabaci* MED genome. All the three *TLP* genes are annotated as thaumatin‐like protein. The complete coding sequences of the three *TLP* genes were manually corrected with their genome or transcriptome dataset. The specific cloning primer pairs were designed by Primer Premier 5.0 software (Table S1, Supporting Information). The LA Taq polymerase (TaKaRa) was used to amplify the products of the three *TLP* genes, which were purified, cloned into a pEASY‐T1 cloning vector (TransGen) and sequenced. The finally obtained full‐length cDNA sequences of *NtTLP*, *TvTLP*, *BtTLP*, and *NtOPR3* were deposited in the GenBank database (GenBank accession nos. PP778349‐PP778352, see Text S1, Supporting Information).

### Phylogenetic Analysis

The BtTLP and TvTLP proteins were used as queries to identify homologs by BLASTp (with “Expect threshold” set at 1E‐15) search in the GenBank database. The top 50 hits in each BLASTp result were selected for phylogenetic tree construction. These sequences were respectively aligned with MAFFT v7.311 using L‐INS‐I option (https://mafft.cbrc.jp/alignment/software). The alignments were used to infer the phylogenetic tree by MrBayes V3.2.7 3 (https://nbisweden.github.io/MrBayes/). For phylogenetic analysis of TLP, the WAG+I+G+F protein substitution matrix was applied. All the phylogenetic trees were displayed and annotated using iTol (https://itol.embl.de/). Last, a tBLASTn search (E‐value cutoff of 1e‐15 and % identity cutoff of >50%) was performed against de novo transcriptomes of *Aleyrodes proletella* (SRR18920704, SRR18920710, SRR18920713, SRR18920719, SRR18920722, and SRR18920723), *Dialeurodes citri* (SRR949617, SRR1015076) and *Aleurocanthus spiniferus* (SRR17330021), as well as de novo genomes of other Aleyrodidae whitefly species, including *Aleuroclava psidii* (SRR16114381), *Aleyrodes shizuokensis* (SRR13162646) and *Singhiella simplex*/*Pealius mori* (ERR3781281), using CLC Genomics Workbench version 22.0.1 with default settings and deposited sequencing data.^[^ ^31^ ^]^ The open reading frame (ORF) of resulting tBLASTn hits were used in a BLASTp search against the nr protein database in NCBI, and those ORFs having the best BLASTp hit with plant TLPs were considered as plant contamination in the raw sequencing data of the whitefly species (Table S2, Supporting Information).

### Bioinformatic Analysis

The detail method to confirm the incorporation of *TvTLP* and *BtTLP* into the *T. vaporariorum* and *B. tabaci* MED genome as previously described.^[^ ^4^, ^31^, ^32^ ^]^ Alignments were sorted with samtools 1.17 with default parameters (https://github.com/samtools/) and visualized with IGV 2.16.0 (https://software.broadinstitute.org/software/igv/). The coverage of each alignment was also calculated by IGV. The signal peptide was predicted using the SignalP 6.0 server (http://www.cbs.dtu.dk/services/SignalP/). Protein‐specific motifs were searched for and analyzed using the SMART server (https://smart.embl.de/). Prediction of protein N‐ and O‐glycosylation sites using the NetNGlyc 1.0 (http://www.cbs.dtu.dk/services/NetNGlyc/) and the NetOGlyc 4.0 (http://www.cbs.dtu.dk/services/NetOGlyc/) servers, respectively. The conversed protein motifs were displayed by the WebLogo 3 program (http://weblogo.threeplusone.com/create.cgi). Alignment of TLPs sequences was performed using CLUSTAL and GENEDOC software.

### Genomic DNA Isolation and Cloning

The Genomic DNA (gDNA) of *T. vaporariorum* adults and *B. tabaci* MED adults was isolated using the TIANamp Genomic DNA Kit (TIANGEN) following manufacturer's instructions, and the gDNA of tobacco was isolated using the Plant Genomic DNA Extraction Kit (GenStar). The quality of the isolated gDNA samples was checked with a NanoDrop 2000c (Thermo Fisher Scientific) and 1.0% agarose gel electrophoresis to ensure the integrity of the DNA. Specific primers were designed using Primer Premier 5.0 based on the *T. vaporariorum* and *B. tabaci* MED genome sequences (Table S1, Supporting Information). The amplified products of the intergenic genomic regions of the *TvTLP* and *BtTLP* were purified and cloned into the pEASY‐T1 vector and sequenced.

### qPCR Analysis

The gene‐specific primers were designed by Primer Premier 5.0 (Table S1, Supporting Information), and qPCR was performed using the QuantStudio 3 real‐time PCR system (Applied Biosystems). The 20 µL PCR reactions contained 0.4 µL of 50 × ROX Reference Dye (TIANGEN), 0.5 µL of each specific primer, 1 µL of cDNA template, 9.5 µL of ddH_2_O, and 10 µL of 2 × SuperReal PreMix Plus (SYBR Green) (TIANGEN). The qPCR reactions were performed with the following protocol: initial denaturation of 94 °C for 3 min, followed by 40 cycles of 95 °C for 15 s, 60 °C for 30 s, and 72 °C for 30 s.

Relative quantification was calculated according to the 2^–ΔΔCt^ method,^[^ ^33^ ^]^ and to accurately analyze the expression of the target genes, the expression data were normalized to the internal gene elongation factor 1 alpha (*EF1‐a*) (GenBank accession no. EE600682) and glyceraldehyde‐3‐phosphate dehydrogenase (*GAPDH*) (GenBank accession no. XM_01 665 5379.1). Three independent biological replicates and four technical replicates were performed for each whitefly sample and tobacco sample.

### dsRNA Synthesis and RNAi Assays

The gene‐specific dsRNA primers of *TvTLP*, *BtTLP* and the negative control of *enhanced green fluorescent protein* (*EGFP*) (GenBank accession no. KC896843) were designed in gene‐specific regions to avoid potential off‐target effects (Table S1, Supporting Information), their specificity was further confirmed by BLASTn against the GenBank database, *T. vaporariorum* and *B. tabaci* genome databases, no hits to any other homologous genes were detected, which further confirming the dsRNA specificity. The dsRNAs were synthesized by the T7 RiboMAX Express RNAi system (Promega) according to the manufacturer's protocols.

An RNAi approach was used to knockdown *TvTLP* and *BtTLP* by feeding the whiteflies dsRNA via artificial diet. Approximately 60 newly emerged *T. vaporariorum* and *B. tabaci* adults were placed separately into the feeding setup, and 0.2 mL of diet solution with 0.5 µg µl^−1^ dsRNA of the target gene was fed to *T. vaporariorum* and *B. tabaci*. After the newly emerged *T. vaporariorum* and *B. tabaci* adults had fed for 24, 48, and 72 h, the RNAi efficacies in knocking down *TvTLP* and *BtTLP* were examined by qPCR.

### Fungi Infection

To obtain fungal infected plants, conidia of *A. alternata*, *B. cinerea* and *C. destructivum* were collected and adjusted to a concentration of ≈10^5^ conidia mL^−1^. The spore suspension was infiltrated into the leaves of *N. tabacum* using a 1 mL needleless syringe.

For entomogenous fungi infecting *T. vaporariorum*, 100 newly emerged adults of *T. vaporariorum* were placed in clip cages attached to transgenic‐EGFP and transgenic‐TvTLP tobacco plants. Tobacco leaves were treated with a microsprayer (0.3 mm needle) calibrated to apply 250 µl of spore suspension per leaf with uniform coverage. As a control, 0.01% Tween‐80 in sterilized water was sprayed.

### Antifungal Activity Assay

To test the growth inhibitory activity of TvTLP against fungi, dilutions of the TvTLP protein (0, 0.3125, 0.625, 1.25, 2.5, and 5 µm) were added to 1.5 mL centrifuge tubes, each containing a spore suspension of ≈10^5^ conidia mL^−1^. Quantitative inhibition of fungal growth was determined by counting of fungal conidia using a hemocytometer after 48 h. Based on the recorded data, IC_50_ values (half‐maximal inhibitory concentration) were calculated. The fungal conidia treated with TvTLP were observed using an oil immersion lens under an inverted microscope (Olympus BX51).

For the fungal spore viability assay, 0.1% resazurin (Sigma) was add to each 96‐well plate containing TvTLP protein dilutions (0, 0.3125, 0.625, 1.25, 2.5, and 5 µm) and spore suspension (≈10^5^ conidia mL^−1^) for 48 h. Color changes were observed after overnight incubation at 28 °C. Each treatment group was replicated three times.

### SYTOX Green (SG) Membrane Permeabilization Assay

The effect of TvTLP on the membrane integrity of *B. bassiana* conidia and germlings were determined using a SYTOX Green nucleic acid stain (Thermo Fisher Scientific). The conidia and germlings of *B. bassiana* were treated with 2.5 µm TvTLP protein for 48 h, and measured after 15 min of exposure to 1 µm SG using fluorescence confocal microscopy (Zeiss LSM710) with an excitation and emission wavelength of 488 and 540 nm, respectively.

### β‐1,3‐Glucanase Assay

For the β‐1,3‐glucanase assay, 100 µL of Reagent 1 (Solarbio) was add to a centrifuge tube contain 100 µL of TvTLP protein dilutions (0, 0.3125, 0.625, 1.25, 2.5, and 5 µm). After incubation at 37 °C for 1 h, 600 µL of Reagent 2 was added and then incubated at 100 °C for an additional 5 min. To observe color changes the reaction solution was transferred to a 96‐well plate for photography. Each treatment group was replicated three times.

### Detection of Reactive Oxygen Species (ROS)

ROS production in *B. bassiana* conidia exposed to different concentrations of TvTLP (0, 0.3125, 0.625, 1.25, 2.5, and 5 µm) was measured every 30 min for 2 h using the ROS indicator dye 2, 7‐dichlorodihydrofluorescein diacetate (DCFH‐DA) (Solarbio), and the final concentration was 10 µm. ROS levels were quantified by measuring the fluorescence in a Spectra M2*^e^* microplate reader (Molecular Devices) (excitation at 488 nm, emission at 525 nm).

### Lipid Strip Assay

PIP Strips (Echelon Biosciences) and Membrane Lipid Strips (Echelon Biosciences) were used following the manufacturer's protocol. Briefly, the PIP Strips and Membrane Lipid Strips were blocked using phosphate‐buffered saline with 0.05% Tween‐20 (PBST) and 3% bovine serum albumin (BSA) (Solarbio) and the solution was gently agitate for 1 h at room temperature (RT). The protein of TvTLP was diluted to 0.5 mg mL^−1^ in blocking buffer and incubated with the PIP Strips and Membrane Lipid Strips for 1 h at RT with gentle agitation. After washing three times with PBST, the PIP Strips and Membrane Lipid Strips were incubated with the anti‐TvTLP antibody (1:3000) at 4 °C overnight, followed by incubation with goat anti‐rabbit horseradish peroxidase‐conjugated secondary antibody (1:5000, CWBIO). Protein dots were visualized using the SuperSignal West Pico Chemiluminescent Substrate (Thermo Fisher Scientific), and the images were captured by a Tanon‐5200 Chemiluminescent Imaging System (Tanon).

### Electrical Penetration Graph (EPG) Assays

The feeding behavior of individual *B. tabaci* on tobacco was recorded using a Giga‐8 direct‐current electrical penetration graphing (DC‐EPG) system (Wageningen University). Briefly, newly emerged *B. tabaci* were treated with dsEGFP or dsBtTLP, and the treated *B. tabaci* were collected and transferred to healthy tobacco plants. A gold wire with water soluble silver conductive glue was used to connect the abdomen of a *B. tabaci* adult to the EPG amplifier. The other electrode was inserted into the soil in which a single tobacco plant had been planted. Then, *B. tabaci* feeding behavior was continuously monitored for 8 h. The recorded EPG waveforms were then analyzed using PROBE V. 3.4 software (Wageningen University). Sixteen replicates were performed for each treatment.

### Transient Expression Analysis

The full‐length of BtTLP lacking a signal peptide was cloned into the plant expression vector Super1300‐GFP and transformed into *Agrobacterium tumefaciens* strain GV3101 by the heat shock. The cultured cells of the recombinant *A. tumefaciens* strains were collected by centrifugation and resuspended in infiltration buffer (10 mm MES, 10 mm MgCl_2_, and 0.1 mm AS) at an OD_600_ of 0.5, and the suspension was infiltrated into four‐week‐old *N. benthamiana* leaves using a 1 mL needleless syringe. Super1300‐GFP empty vector and Bax (a mouse pro‐apoptotic protein which elicits plant hypersensitive response) were used as the negative and positive controls, respectively. Phenotypic changes in the injected leaves were checked for after 5 days.

For the subcellular localization assay, *A. tumefaciens* strains GV3101 carrying pBin‐GFP empty vector and pBin‐GFP‐BtTLP were collected and resuspended in infiltration buffer at an OD_600_ of 0.5, and the suspension was infiltrated into the four‐week‐old *N. benthamiana* leaves using a 1 mL needleless syringe. After 48 h of infiltration, images were captured by laser confocal fluorescence microscopy (Zeiss LSM710).

### Heterologous Expression and Protein Purification

The coding sequences of *NtTLP*, *TvTLP*, and *BtTLP* were cloned into the pET28a vector, and the recombinant vectors were transformed into *Escherichia coli* BL21 (TransGen). Single positive transformed colonies were selected and sequenced. The corrected single positive transformed colony of each gene was then incubated overnight at 37 °C in 5 mL LB medium (containing 100 µg mL^−1^ kanamycin). One milliliters of overnight incubated LB medium was added to 100 mL of fresh LB medium (containing 100 µg mL^−1^ kanamycin) and incubated at 37 °C. The sopropyl‐β‐dthiogalactopyranoside (IPTG) solution (1 mm, Solarbio) was added to LB culture when OD_600_ of culture reached 0.6. The incubation LB medium was grown at 37 °C, 200 rpm for 3 h, and then harvested by centrifugation at 4000 × *g* for 5 min at 4 °C. The pellets were broken with supersonic waves in 3 mL of phosphate‐buffered saline (PBS) buffer. Cell debris was removed by centrifugation for 15 min at 4000 × *g* at 4 °C and the supernatant was collected. The recombinant proteins were purified using the Amicon Pro Purification System (Millipore). Briefly, proteins were eluted with buffer A (50 mm Tris‐HCl, 200 mm NaCl, and imidazole gradient from 50 to 250 nm, pH 8.3). To remove imidazole, each elution passed through a Hi‐Trap desalting column (GE Healthcare) with buffer B (50 mm tris‐HCl, pH 8.3). Finally, ultrafiltration of the recombinant proteins with a 30 kDa cutoff Amicon Ultra‐0.5 Device (Millipore). The final purified recombinant proteins were verified by SDS‐PAGE and stained with Coomassie staining.

### Antibody Preparation and Western Blot

The specific amino acid fragment of TvTLP (^191^TGDKCKKSDCSADVNAV^207^) and BtTLP (^160^DNHNTREKCPPSNWSRV^176^) were used for antibody synthesis (Pujian Biotech). In order to avoid non‐specificity of the antibody, a BLASTp search (E‐value threshold with 1E‐05) of the designed amino acid fragment in the GenBank (https://www.ncbi.nlm.nih.gov/), the *T. vaporariorum* and *B. tabaci* genomes were blasted, and no hits to *N. tabacum* proteins were detected, confirming the specificity of the selected amino acid fragment. Other specific antibodies targeting insect β‐actin (Abcam) and plant actin (ABclonal) were commercially purchased. The protein level of the target proteins were determined by western blot.

To detect the BtTLP protein in *B. tabaci* adults, *B. tabaci*‐infested tobacco leaves and uninfested tobacco leaves, western blot was performed using purified BtTLP polyclonal antibody. Approximately 10000 *B. tabaci* were released to feed on tobacco plant for 48 h, then the adults and eggs were removed from the leaves. Protein samples (*ca* 30 µg protein extracted from *B. tabaci* adults and tobacco leaves) were isolated using SDS‐PAGE and transferred onto PVDF membranes (Merck 560 Millipore). The PVDF membranes were then blocked with blocking buffer containing BSA at 25 °C for 1 h and incubated with the appropriate primary antibody (1:3000) at 4 °C overnight, followed by incubation with goat anti‐rabbit horseradish peroxidase conjugated secondary antibody (1:5000, CWBIO). Protein bands were visualized using the SuperSignal West Pico Chemiluminescent Substrate (Thermo Fisher Scientific), and images were captured by the Tanon 5200 Chemiluminescent Imaging System (Tanon). Densitometric analysis of the protein bands was performed using ImageJ v.1.51 software (http://rsbweb.nih.gov/ij/), and the relative band intensities were calculated based on densitometric ratios between target proteins and internal controls.

### Immunostaining Assay

The tobacco tissues, including leaf, stem and root, were dehydrated through a graded series of ethanol concentrations ranging from 10% to 100%. Dehydrated samples were then embedded in London Resin White (Sigma‐Aldrich). Sections of 4 µm thickness were cut from the resin blocks with a microtome (Leica RM2016) and placed on adhesive glass slides for immunofluorescence assays. The slides were blocked with 1% goat serum for 3 h at room temperature and incubated overnight at 4 °C with the anti‐NtTLP antibody diluted 1:1000 in PBST. After rinsing five times in PBST, the samples were incubated with goat anti‐rabbit IgG conjugated to Alexa 555 (Abcam, 1:200) as a secondary antibody for 1 h at room temperature, followed by another five rinses in PBST. Sections were imaged using a Zeiss LSM710 confocal microscope.

The salivary glands, midguts, male and female gonads were dissected from *T. vaporariorum* and *B. tabaci* adults. The samples were then subjected to fixation using a 4% solution of paraformaldehyde for 1 h at room temperature and washed three times with PBST. Then, the samples were incubated in PBST with 1% BSA for 3 h at room temperature and subsequently incubated with the BtTLP antibody (1:3000) at 4 °C overnight. After five rinses in PBST, samples were incubated with goat anti‐rabbit IgG conjugated to Alexa 555 (Abcam, 1:200) as secondary antibody for 1 h at room temperature. Then, the samples were again washed five times in PBST, and mounted with Fluoroshield Mounting Medium with DAPI (Abcam). The images were captured using a Zeiss LSM710 confocal microscope using wavelength (DAPI: excitation at 353 nm, emission at 465 nm; TvTLP and BtTLP: excitation at 545 nm, emission at 572 nm).

### Transgenic Tobacco Plants Construction

To create RNAi‐mediated transgenic tobacco lines, the hairpin RNA expression vector (pEXT06‐RNAi) was introduced into tobacco (*N. tobacco* K326), for which the specific fragment of *TvTLP* and *BtTLP* were cloned using specific primers (Table S1, Supporting Information). The purified PCR products were tandemly inserted into KpnI‐SaII‐cut of pEXT06 and reversely tandemly inserted into Bam HI‐SacI‐cut of pEXT06.

For the creation of overexpression tobacco lines, the full‐length of *NtTLP*, *BtTLP*, and *NtOPR3* were cloned with specific primers (Table S1, Supporting Information). The purified PCR products were inserted into KpnI‐PstI‐cut of BGV008.

To create knockout transgenic tobacco lines, the CRISPR/Cas9 system was used to knockout *NtTLP* and *NtOPR3* from *N. tabacum* K326. The *koNtTLP and koNtOPR3* lines were generated via the custom service of CRISPR RGEN tool Cas‐Designer (http://www.rgenome.net/cas‐designer/). Briefly, the specific target of *NtTLP* (5′‐CCTCGACGGTTCCGGCGGAATGG‐3′) and *NtOPR3* (5′‐CCAGGGCGCCTGTCCGACTCAAA‐3′) was predicted using CRISPR RGEN tool Cas‐Designer (http://www.rgenome.net/cas designer/). The potential off‐target effects of all the sgRNA target sequences were eliminated by searching in the GenBank database (https://www.ncbi.nlm.nih.gov/) and the CRISPR RGEN Cas‐OFFinder tool (http://www.rgenome.net/casoffinder/). Then, they were ligated into the single guide RNA (sgRNA) expression cassettes by overlapping PCR. The PCR product was then cloned into the CRISPR/Cas9 vector.

Both the recombinant pEXT06‐RNAi vector, the BGV008 overexpression vector and the CRISPR/Cas9 vector were transformed into *A. tumefaciens* EHA105 by electroporation. Tobacco transformation was performed using the *A. tumefaciens* through callus inoculation and plant regeneration.

The RNAi transgenic tobacco lines and the knockout transgenic tobacco lines were confirmed by PCR using specific primers and the genomic DNA as template, followed by Sanger sequencing. The overexpressing transgenic tobacco lines were confirmed via western blot or qPCR. Two independent T2 RNAi lines, overexpression lines and homozygous knockout lines were used for subsequent experiments.

### Biological Assays

First the impact of RNAi targeting *TvTLP* via artificial diet on *T. vaporariorum* fungal resistance was assessed. For this, *TvTLP*‐silenced *T. vaporariorum* adults were released into tubes with or without *B. bassiana* and *M. robertsii* (≈10^5^ conidia mL^−1^) and their survival rate and hatching success were recorded.

For RNAi via transgenic tobacco plants, 100 newly emerged adults of *T. vaporariorum* were placed in clip cages attached to transgenic‐*EGFP* and transgenic‐*TvTLP* tobacco plants. Tobacco leaves were treated with a microsprayer (0.3 mm needle) calibrated to apply 250 µl of spore suspension per leaf with uniform coverage, 0.01% Tween‐80 sterilized water was sprayed as a control. Again, survival rate and hatching success of *T. vaporariorum* were recorded.

To investigate the impact of RNAi targeting *BtTLP* on *B. tabaci* survival on tobacco and artificial diet, *BtTLP*‐silenced *B. tabaci* were released into clip cages attached to a tobacco plant and tubes respectively. Ten newly emerged female adults of *B. tabaci* MED were kept in each clip cage and tube. The number of surviving adults in the cages and tubes was recorded for 7 days. Six independent biological replicates were conducted for each treatment.

The performance of *B. tabaci* on RNAi and overexpression *BtTLP* transgenic tobacco lines, as well as knockout and overexpression *NtOPR3* transgenic tobacco lines was assessed. Ten newly emerged female adults of *B. tabaci* MED were released into clip cages attached to the tobacco plants. The number of surviving adults and eggs were recorded after 7 days.

### Measuring the Levels of Salicylic Acid (SA), Jasmonic Acid (JA) and JA‐Isoleucine (JA‐Ile)

Leaf samples were harvested and ground in liquid nitrogen, and then 100 mg of ground powder was diluted in 2 ml MeOH with 1 ppm of D4‐SA, D6‐JA, and D6‐JA‐Ile (CDN Isotopes) as internal standards. The samples were centrifuged for 15 min at 12000 rpm at 4 °C, the supernatant was then collected and evaporated under nitrogen, and the residue was dissolved in 1 mL ammonia solution (5%, v/v). The content of extract samples was measured by the ultra‐performance liquid chromatography–tandem mass spectrometry (UPLC‐MS/MS) (Waters). Each treatment was replicated three times.

### Protein‐Protein Interaction (PPI) Assays

For the *N. tabacum* cDNA library construction, the samples of tobacco leaves infested with *B. tabaci* for 0, 1, 2, and 3 days were sent to Zoonbin Biotechnology. In the yeast two‐hybrid (Y2H) screening assay, the full‐length of BtTLP without the signal peptide sequence was constructed into the pGBKT7 vector. The recombinant vectors and the *N. tabacum* cDNA library were then co‐transfected into the yeast strain Y2H Gold. Y2H screen was performed using the MATCHMAKER Gold Yeast Two‐Hybrid System (Clontech) following the manufacturer's protocol.

For immunoprecipitation‐mass spectrometry (IP‐MS) assay, the BtTLP without signal peptide was cloned into the pBin‐GFP vector. The recombinant plasmid was transformed into *A. tumefaciens* GV3101, and then used to infiltrate *N. benthamiana* leaves. Total proteins were extracted from *N. benthamiana* leaves after 48 h infiltration. IP‐MS was performed with Anti‐GFP‐tag mAb‐Magnetic Agarose (MBL). Briefly, 30 µL agarose beads were washed with 1 mL protein extraction buffer. Then, 1 mL of *N. benthamiana* total protein was added and incubated with rotation at 4 °C for 1 h. The agarose beads were washed 5 times with 1 mL protein extraction buffer, and then the proteins were eluted with 1 × SDS loading buffer. Subsequently, the proteins were detected by western blot using GFP antibody (MBL). A Q‐Exactive HF‐X Easy nLC1200 mass spectrometer system (Thermo Fisher Scientific) was used for liquid chromatography‐tandem mass spectrometry (LC‐MS/MS) at Shanghai Bioprofile Technology. The acquired mass spectrometric data were pre‐analyzed using MaxQuant 1.6.1.0 and then annotated to search the UniProt Protein Database (https://www.uniprot.org/). Candidate proteins that showed specific immunoprecipitation in the pBin‐GFP‐BtTLP groups but not in the control of pBin‐GFP were selected.

In the Y2H point‐to‐point verification assay, the full‐length of BtTLP lacking a signal peptide was fused to GAL4 DNA‐binding domain (BD), and NtOPR3 was fused to the GAL4 activation domain (AD). Then, the recombinant vector and control were co‐transfected into the yeast strain Y2H Gold, and incubated on the double dropout (DDO) medium (SD/‐Leu/‐Trp) (TaKaRa) at 30 °C for 3 days. Then, the monoclonal colonies were selected and spotted on quadruple dropout media (QDO) medium (SD/‐Trp/‐Leu/‐His/‐Ade) (TaKaRa).

In the co‐immunoprecipitation (Co‐IP) assay, BtTLP and NtOPR3 were cloned into pBin‐GFP and Super1300‐FLAG vector. Both recombinant vectors were co‐expressed in four‐week‐old *N. benthamiana* leaves. Total proteins were extracted from *N. benthamiana* leaves after 48 h infiltration. Co‐IP was performed with Anti‐GFP‐tag mAb‐Magnetic Agarose (MBL). Briefly, 30 µL agarose beads were washed with 1 mL protein extraction buffer. Then, 1 mL of *N. benthamiana* total protein was added and incubated with rotation at 4 °C for 1 h. The agarose beads were washed 5 times with 1 mL protein extraction buffer, and then the proteins were eluted with 1 × SDS loading buffer. Subsequently, the proteins were detected by western blot using anti‐GFP antibody (MBL) and anti‐Flag antibody (ABclonal).

In the bimolecular fluorescence complementation (BiFC) assay, BtTLP was cloned into the cYFP vector, NtOPR3 was cloned into nYFP vector. The two recombinant vectors were co‐transfected into *A. tumefaciens* GV3101 as described above. Then, the *A. tumefaciens* transfected with recombinant vectors and corresponding empty vectors were co‐infiltrated into *N. benthamiana* leaves for 48 h. The YFP fluorescence was captured under a Zeiss LSM710 confocal microscope.

In the luciferase complementation (LUC) assay, BtTLP was cloned into the nLUC vector and NtOPR3 was cloned into the cLUC vector. The recombinant vectors and the corresponding empty vectors were co‐transformed into *A. tumefaciens* GV3101, respectively, and then co‐infiltrated into different areas of the same *N. benthamiana* leaf. After 48 h infiltration, 1 mm luciferin (BioVision) solution sprayed onto the infiltrated leaves areas. The luciferase activity was detected using a low light cooled CDD imaging apparatus ChemiScope 6000 (Clinx Science Instruments).

### BtTLP Destabilization of NtOPR3 Assay

The recombinant vectors BtTLP‐GFP and NtOPR3‐FLAG were transfected into *A. tumefaciens* GV3101, which were used to infiltrate four‐week‐old *N. benthamiana* leaves. The 26S proteasome inhibitor 100 µm MG132 (Selleck) was infiltrated into *N. benthamiana* leaves after 24 h. Total proteins were extracted from *N. benthamiana* leaves after 48 h infiltration. The protein levels were detected by western blot analysis.

### Statistical Analysis

All the data were analyzed using the IBM SPSS Statistics (ver. 26.0) software (IBM Corp.). Data were shown as the means ± SEM. The data statistical significance was determined using one‐way ANOVA with Tukey's test (*P < 0.05, **P < 0.01, ***P < 0.001).

## Conflict of Interest

The authors declare no conflict of interest.

## Author Contributions

Y.H., C.G., and Z.Y. contributed equally to this work. Y.H., C.G., X.Z., T.C.J.T., Z.G., and Y.Z. designed the research. Y.H., C.G., Z.Y., H.H., T.T., X.Y., W.X., S.W., Q.W., and Z.G. performed the experiments. Y.H., C.G., Z.Y., and Z.G. analyzed the data; Y.H., C.G., X.Z., W.D., T.C.J.T., Z.G., and Y.Z. wrote and revised the manuscript.

## Supporting information



Supporting Information

Supplemental Table 2

Supplemental Table 3

Supplemental Table 4

Supplemental Text 1

## Data Availability

The authors declare that the data supporting the findings of this study are available within the paper and its Supplementary Information. The full‐length cDNA sequences of all the cloned genes in this study have been deposited in the GenBank database under accession nos. PP778349‐PP778352.
