# Supplementary material for: Functional Divergence of Plant‐Derived Thaumatin‐Like Protein Genes in Two Closely Related Whitefly Species
Source: Adv Sci (Weinh). 2025 Feb 28;12(16):2502193. doi: 10.1002/advs.202502193 (PMC12021119; doi:10.1002/advs.202502193)
Supplement: Supplementary file 1 — Supporting Information [file ADVS-12-2502193-s002.pdf]

ADVANCED SCIENCE
Open Access

Supporting Information

for *Adv. Sci.*, DOI 10.1002/advs.202502193

Functional Divergence of Plant-Derived *Thaumatin-Like Protein* Genes in Two Closely Related Whitefly Species

Yuan Hu, Cheng Gong, Zezhong Yang, Haolin Han, Tian Tian, Xin Yang, Wen Xie, Shaoli Wang, Qingjun Wu, Xuguo Zhou, Ted C. J. Turlings, Zhaojiang Guo* and Youjun Zhang**

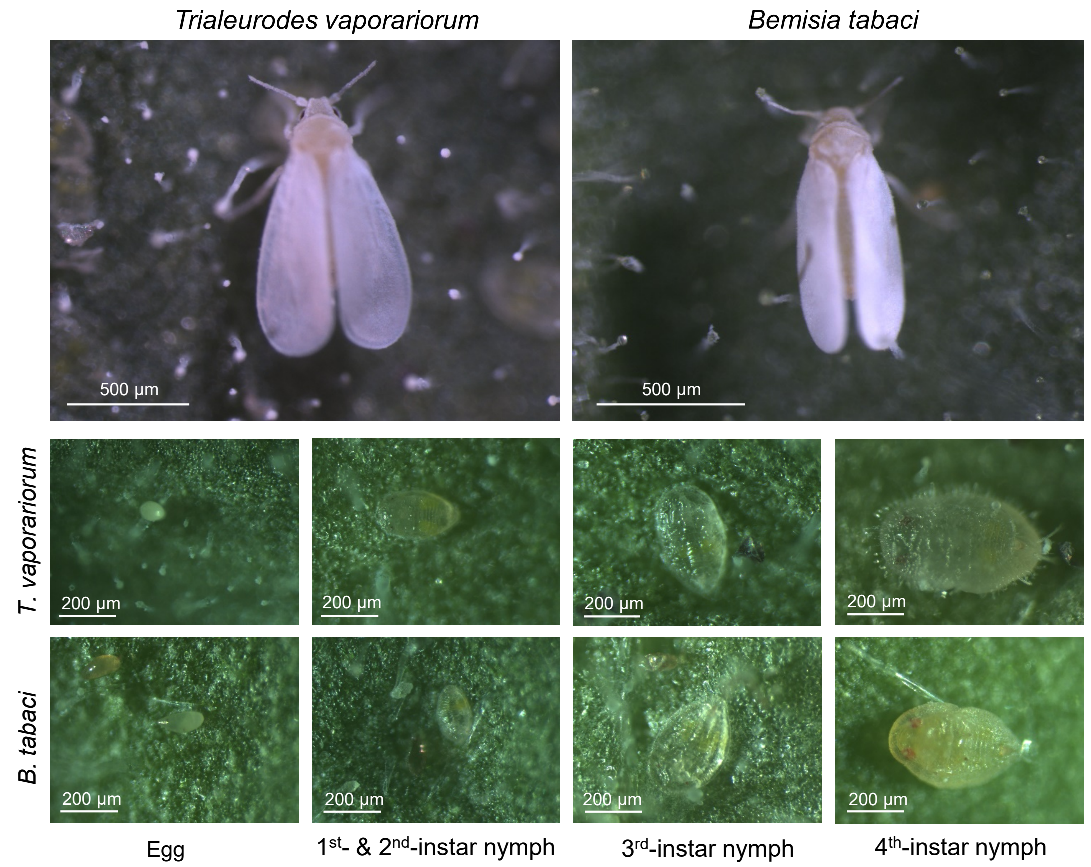

Figure S1. Phenotypes of *Trialeurodes vaporariorum* and *Bemisia tabaci* at all developmental stages on healthy tobacco leaves.

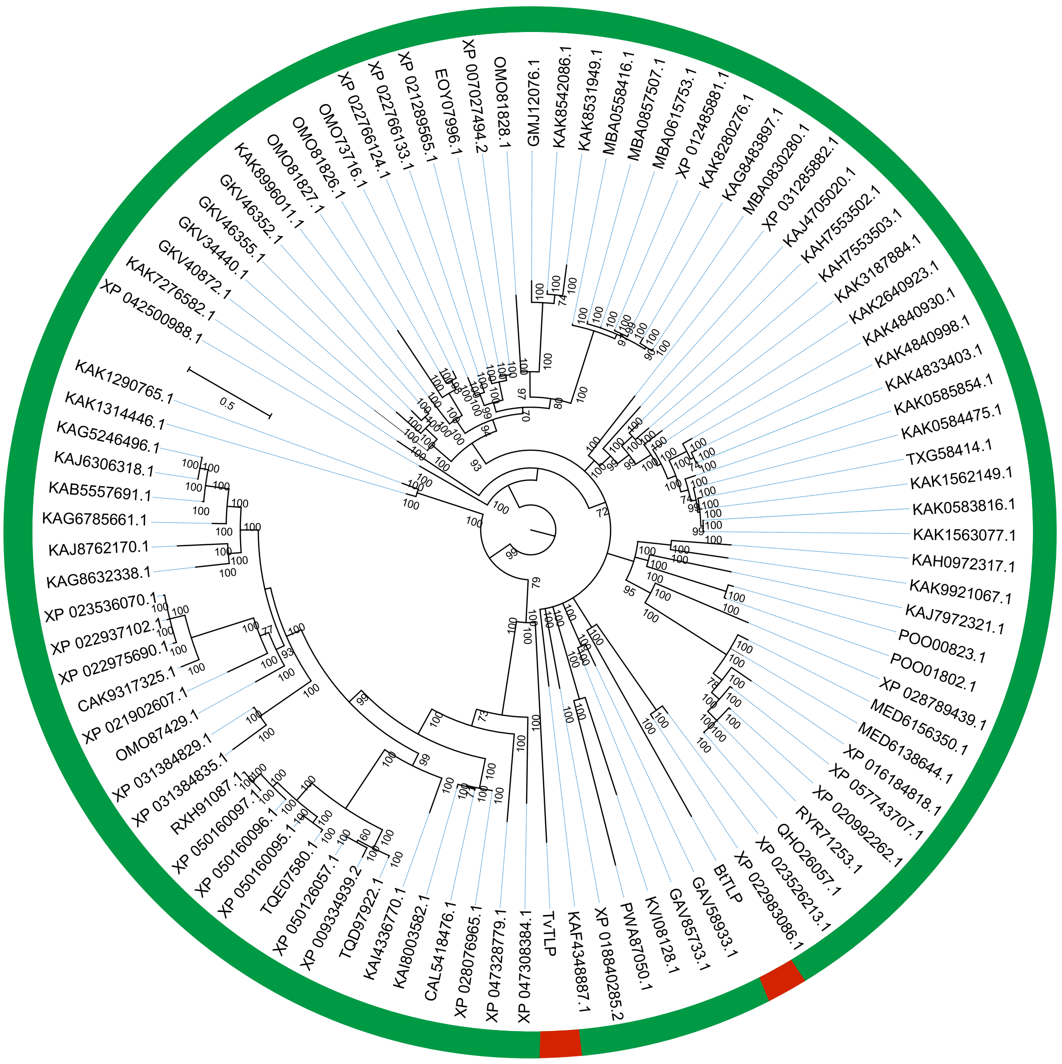

Figure S2. The Bayesian-based phylogenetic analysis of TvTLP and BtTLP. The red color indicates *T. vaporariorum* TLP and *B. tabaci* TLP, and green color indicates plant TLP. The protein sequences that correspond to the accession numbers in the tree were extracted from the GenBank database. The scale bar indicates 0.5 amino acid substitutions per site.

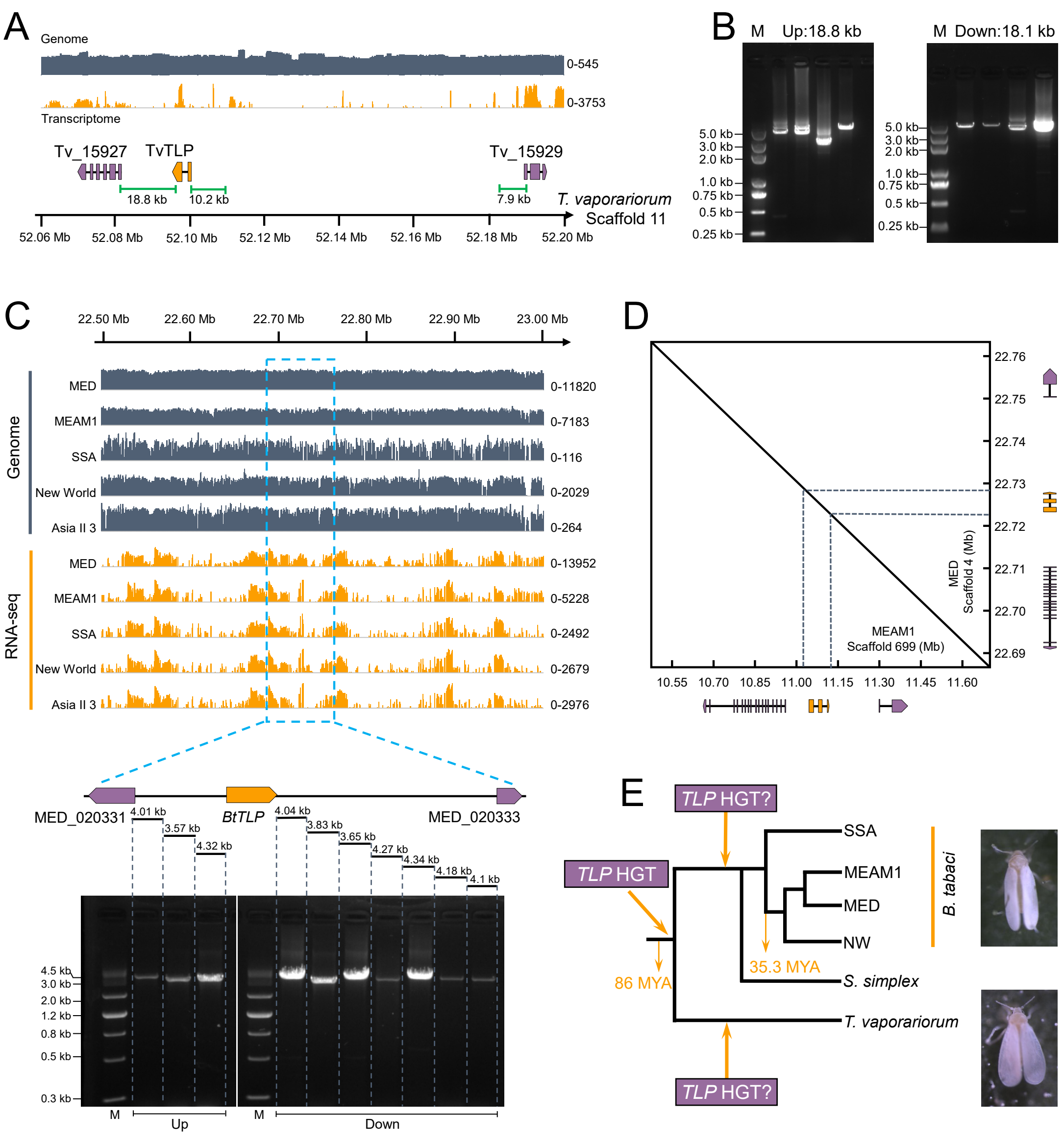

Figure S3. Evidence for the horizontal transfer of *TvTLP* into *T. vaporariorum* genome and *BtTLP* into *B. tabaci* genome. A) Genomic location of *TvTLP* in *T. vaporariorum*. Illumina DNA-read coverage plot and Illumina RNA-seq read coverage plot from *T. vaporariorum* are displayed, the sequence depths are shown by the numbers on the right of the coverage plots. PCR cloning of genomic fragments are indicated in green. B) Genome fragments cloned from *T. vaporariorum* by overlapping PCR. Up, Tv_15927-TvTLP genome fragment; Down, TvTLP-Tv_15929 genome fragment. C) Genomic location of *BtTLP* in different *B. tabaci* cryptic species and genome fragments cloned

from *B. tabaci* MED by overlapping PCR. Up, MED_020331-BtTLP genome fragment; Down, BtTLP-MED_020333 genome fragment. DNA-read coverage plot and Illumina RNA-seq read coverage plot from different *B. tabaci* cryptic species are displayed, the sequence depths are shown by the numbers on the right of the coverage plots. D) Genome synteny of the *BtTLP* and their respective two surrounding genes in *B. tabaci* MED and *B. tabaci* MEAM1. The black diagonal line indicates the similarity of two genome regions by more than 95%. Genome fragments cloned from *B. tabaci* MED are indicated in orange. E) Graphic depiction of the proposed evolutionary history of *TLP* genes in different cryptic species of *T. vaporariorum* and *B. tabaci*. The event of *TLP* gene transfer before *B. tabaci* divided from *T. vaporariorum* (86 MYA) and the event after *B. tabaci* divided into different cryptic species (35.3 MYA) are denoted. Abbreviation: MED: *B. tabaci* MED; MEAM1: *B. tabaci* MEAM1; SSA: *B. tabaci* SSA; NW: *B. tabaci* New World. Asia II 3: *B. tabaci* Asia II 3; *S. simplex*, *Singhiella simplex*.

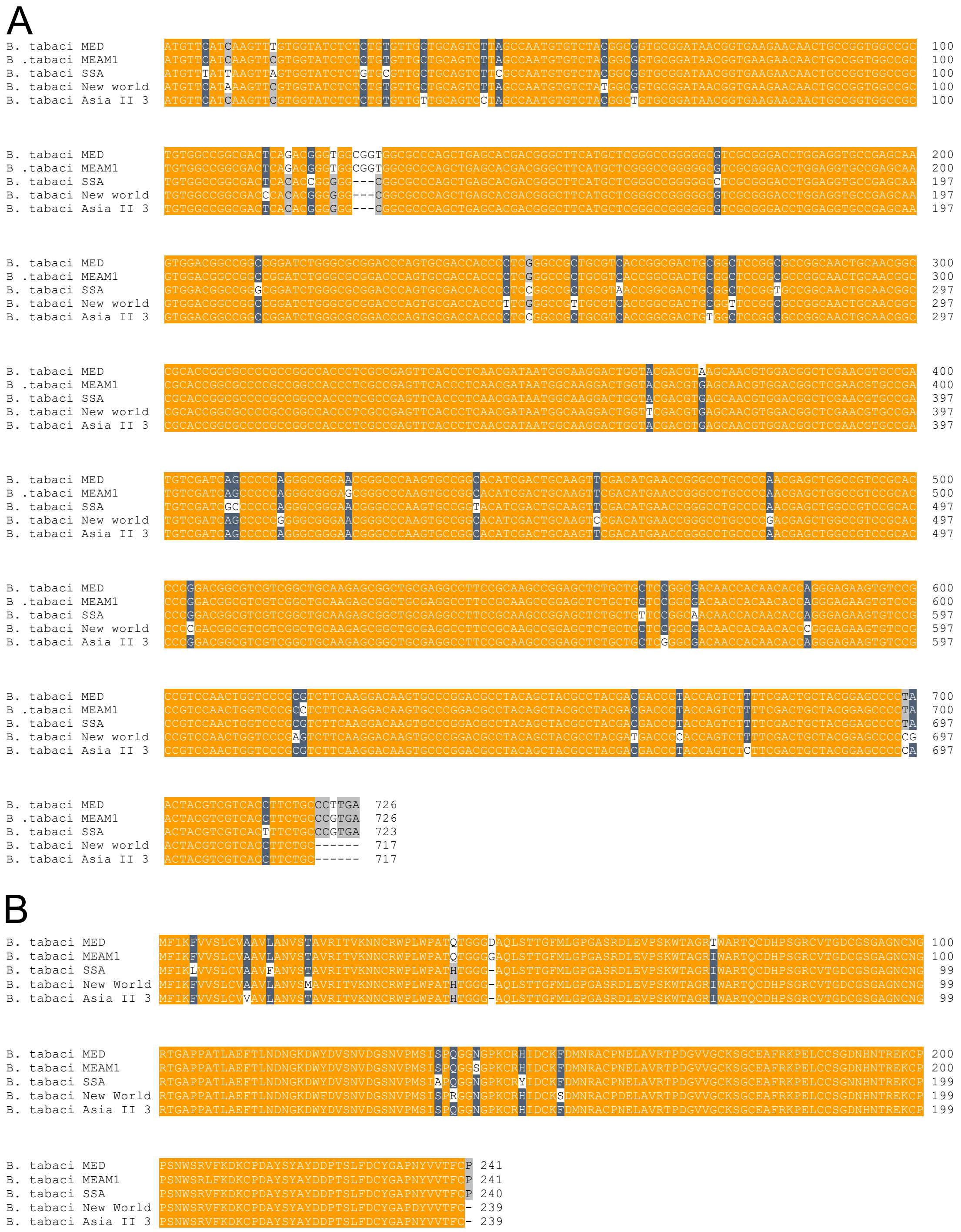

Figure S4. Sequence alignment of BtTLP in different cryptic species of *B. tabaci* (MED, MEAM1, SSA, New World and Asia II 3). A) Nucleic acid sequence alignment of *BtTLP*. B) Amino acid sequence alignment of BtTLP.

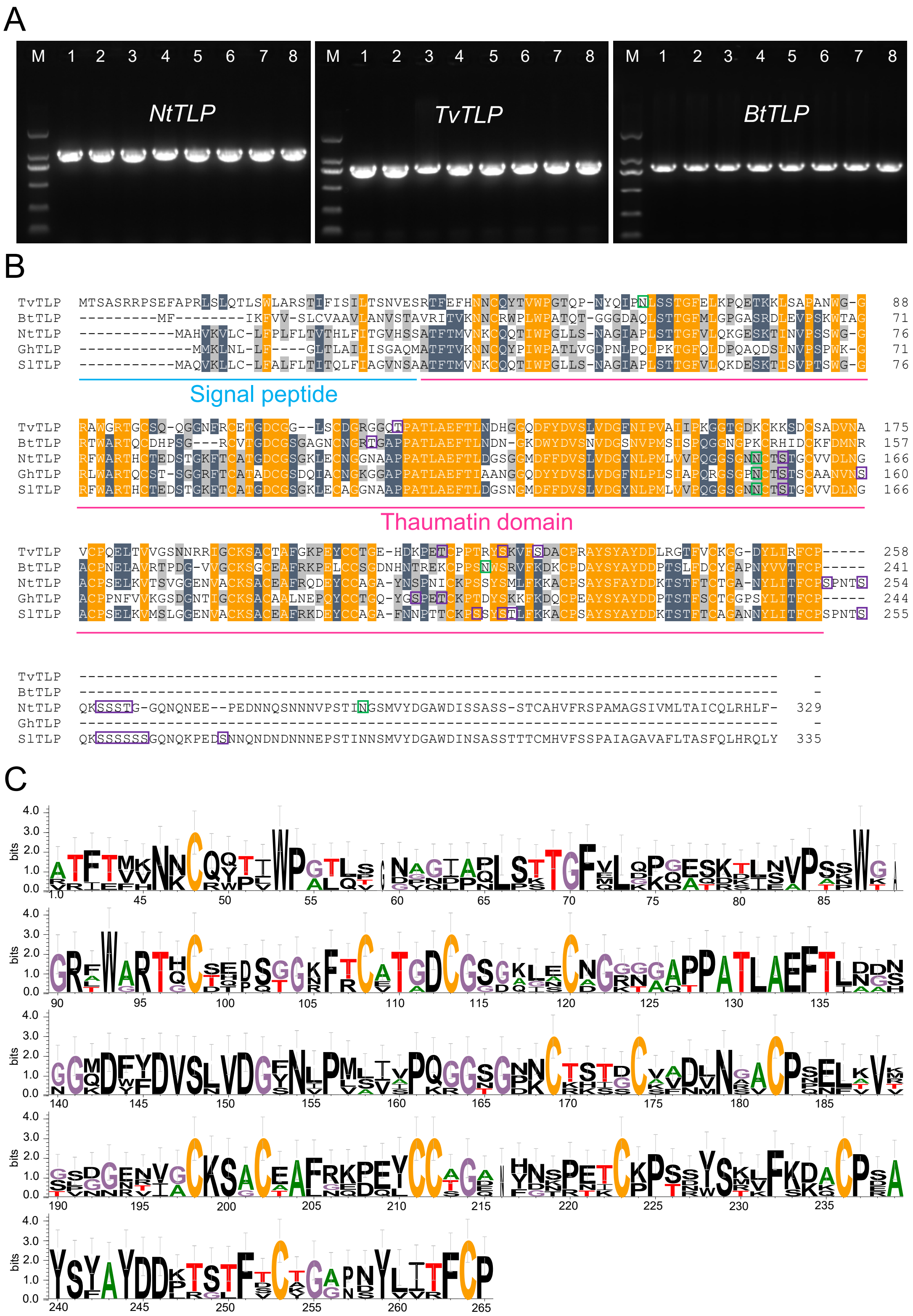

Figure S5. Cloning and sequence alignment of *NtTLP*, *TvTLP* and *BtTLP*. A) The *TLP* gene was cloned from *N. tabacum*, *T. vaporariorum* and *B. tabaci* MED. Lanes 1-8, PCR products. M, marker (from top to bottom: 2,000 bp, 1,000 bp, 750 bp, 500 bp, 250

bp, 100 bp). B) Amino acid sequence alignment of TvTLP, BtTLP and their plant homologs TLP proteins. The signal peptide is underlined in blue, the conservative thaumatin domain is underlined in red, the putative N-glycosylation sites are indicated by the green rectangle and O-glycosylation sites by the purple rectangles. C) WebLogo plots highlight amino acid conservation of TvTLP, BtTLP and their plant homologs TLP proteins. The sixteen conserved cysteine residues are shown in orange.

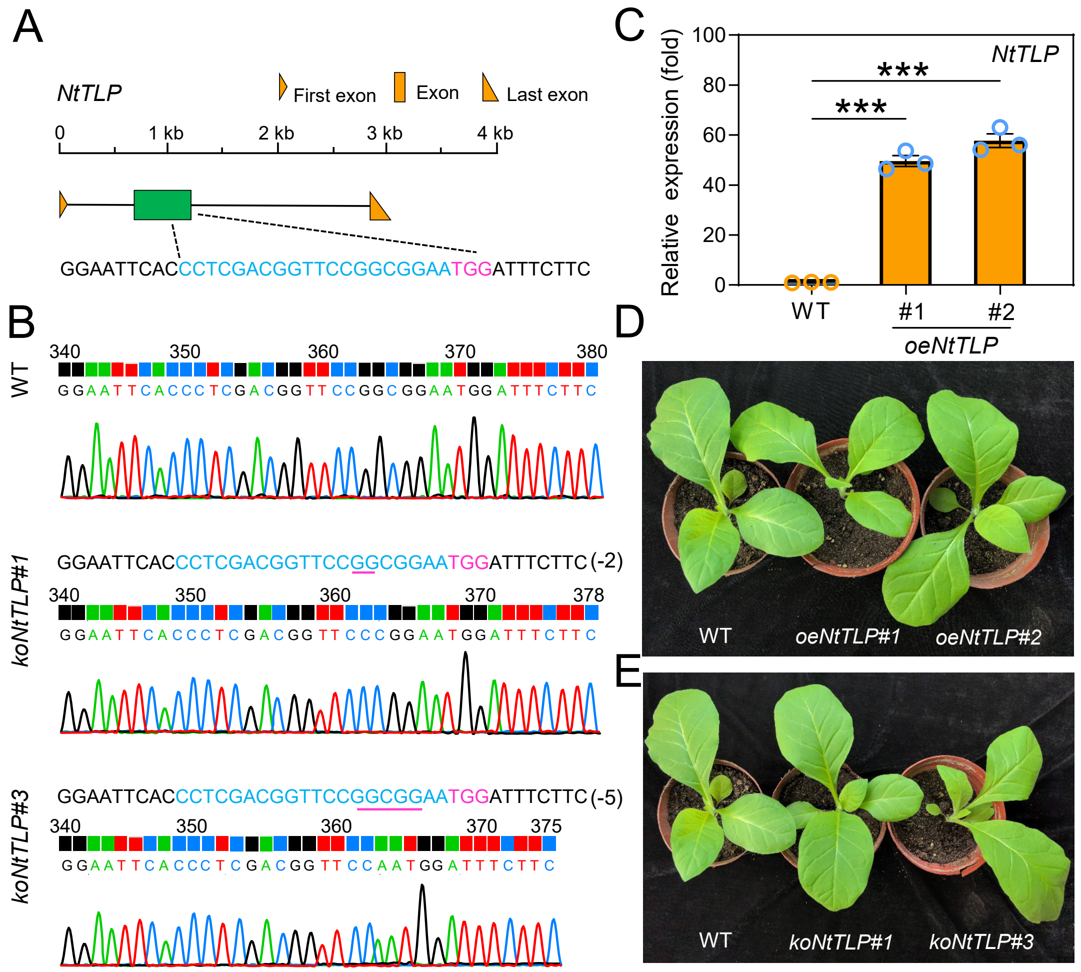

Figure S6. Analysis of the editing sequence and the transcript level of *NtTLP* in transgenic tobacco plants. A) The designed sgRNA-targeted sites and sequences in exon 2 (green boxes) of *NtTLP*. Exons are shown as orange boxes and green triangle, spaces between two exons indicate the introns. The sgRNA of target gene is highlighted in blue, the PAM sequences are marked in pink. B) Sanger sequencing in WT and two independent mutations of *koNtTLP* plants. The locations of CRISPR/Cas9-induced 2-bp deletion (GG) of *koNtTLP#1* and 5-bp deletion (GGCGG) of *koNtTLP#3* within exon 2 of *NtTLP* are marked by pink lines. C) The transcript levels of *NtTLP* in two *NtTLP* overexpressing (*oeNtTLP#1*, *oeNtTLP#2*) transgenic tobacco plants were determined by qPCR. D) Phenotypes of WT and the two *NtTLP* overexpressing

(*oeNtTLP#1*, *oeNtTLP#2*) transgenic tobacco plants. E) Phenotypes of WT and the two *NtTLP* mutant (*koNtTLP#1*, *koNtTLP#3*) transgenic tobacco plants. Date are means ± SEM, $n = 3$ (C) biologically independent samples, $***P < 0.001$, one-way ANOVA with Tukey's test was used for comparison.

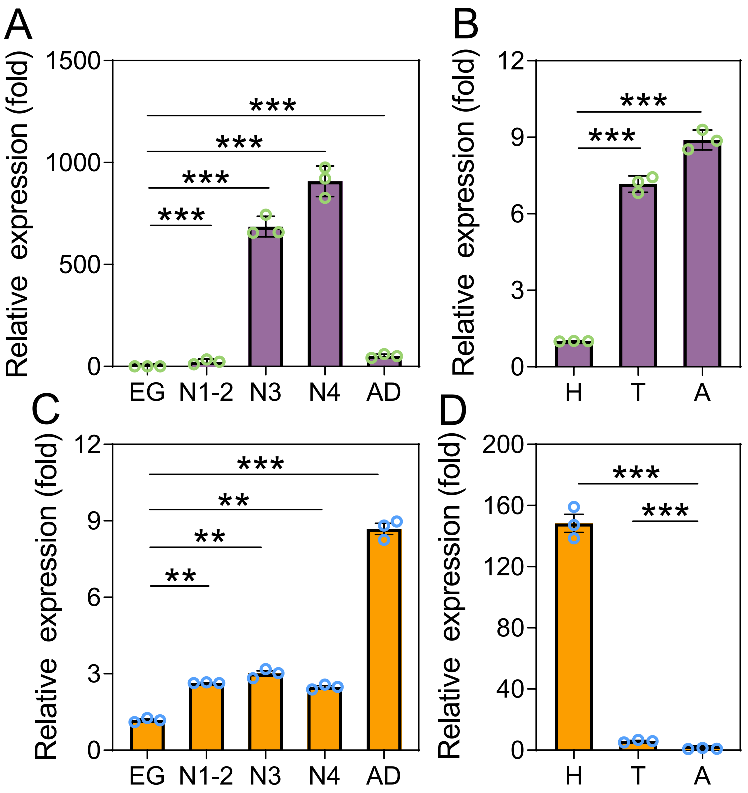

Figure S7. Spatio-temporal expression profiling of TvTLP and BtTLP. A-B) The relative expression levels of *TvTLP* in eggs (EG), first- and second-instar nymphs (N1-2), third-instar nymphs (N3), fourth-instar nymphs (N4), adults (AD), heads (H), thoraxes (T), and abdomens (A) from *T. vaporariorum* adults. The relative expression level (fold change) was calculated based on the lowest measured expression value (Developmental stages: EG; Tissues: H), which was arbitrarily assigned a value of 1. C-D) The relative expression levels of *BtTLP* in eggs (EG), first- and second-instar nymphs (N1-2), third-instar nymphs (N3), fourth-instar nymphs (N4), adults (AD), heads(H), thoraxes (T), and abdomens (A) from *B. tabaci* adults. The relative expression level (fold change) was calculated based on the lowest measured expression value (Developmental stages: EG; Tissues: A), which was arbitrarily assigned a value of 1. Date are means ± SEM, $n = 3$ (A-D) biologically independent samples, $^{**}P < 0.01$, $^{***}P < 0.001$, one-way ANOVA with Tukey's test was used for comparison.

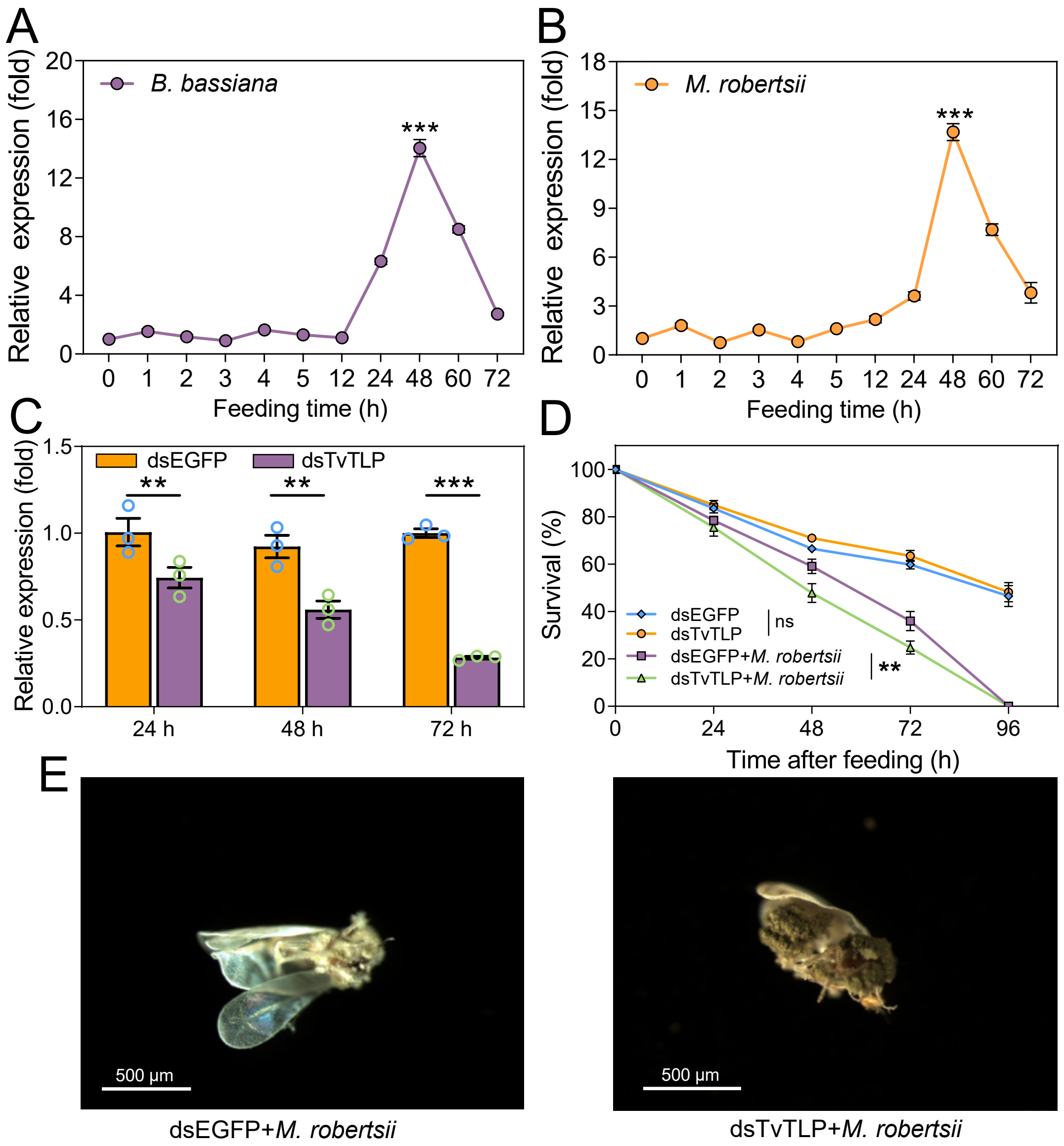

Figure S8. Effect of *TvTLP* silencing on *T. vaporariorum* performance. A-B) The transcript levels of *TvTLP* after *T. vaporariorum* feeding on artificial diet with *B. bassiana* (A) and *M. robertsii* (B) spore suspension for 0 h, 1 h, 2 h, 3 h, 4 h, 5 h, 12 h, 24 h, 48 h, 60 h and 72 h were determined by qPCR. C) The transcript levels of *TvTLP* in *T. vaporariorum* at 24 h, 48 h and 72 h post-RNAi were determined by qPCR, respectively. D) Survival rate of *T. vaporariorum* adults that were fed either dsEGFP or dsTvTLP, with or without *M. robertsii*. E) Typical *M. robertsii*-covered cadavers of dsEGFP and dsTvTLP treated *T. vaporariorum* adults. Date are means ± SEM, n = 3 (A, B) biologically independent samples, $^{**}P < 0.01$, $^{***}P < 0.001$, one-way ANOVA with Tukey's test was used for comparison.

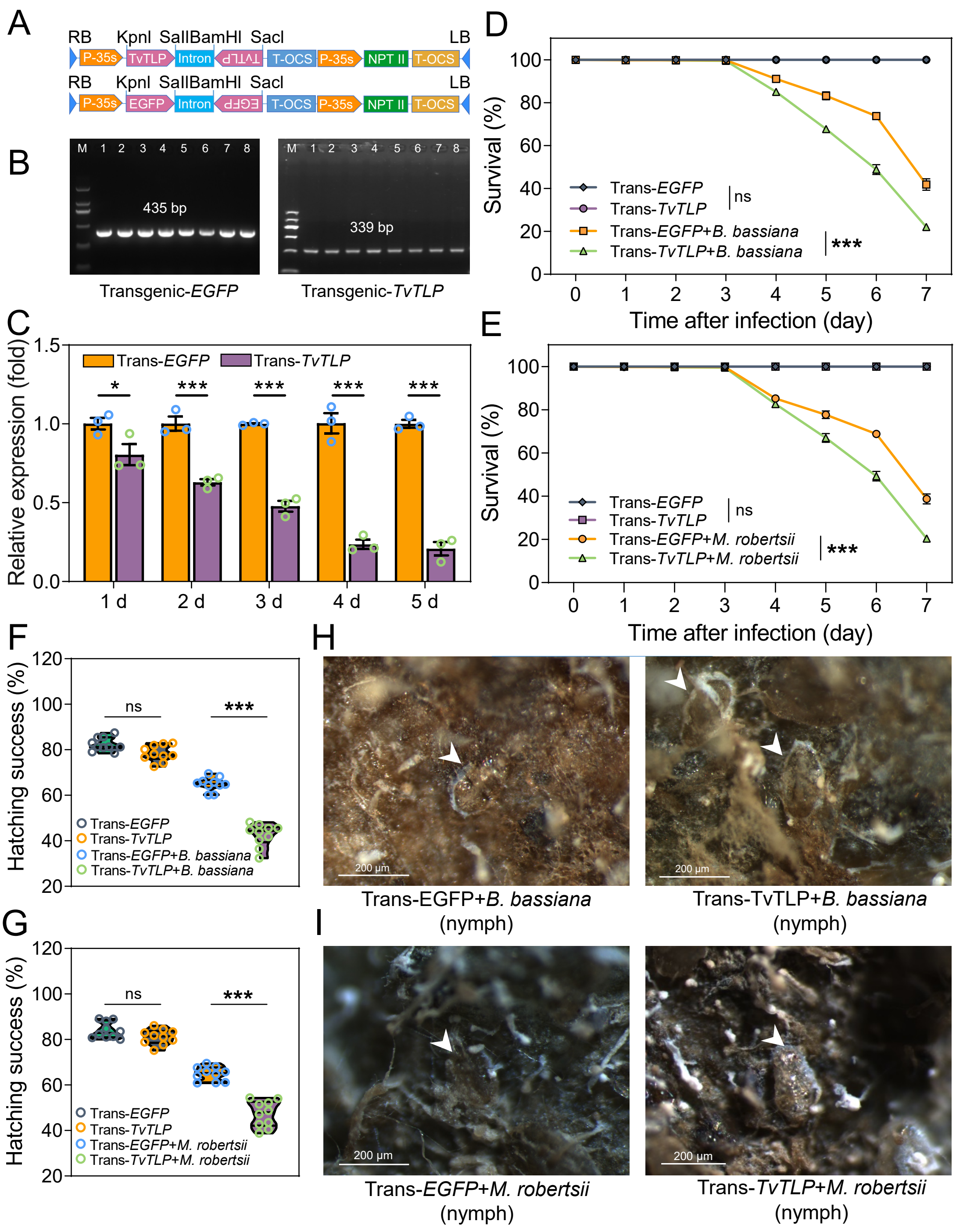

Figure S9. Effect of *TvTLP* silencing on *T. vaporariorum* nymph performance. A) The diagram of RNAi vector construction used for tobacco transformation. B) PCR products amplified from transgenic-*EGFP* and transgenic-*TvTLP* tobacco leaves. M, marker (from top to bottom: 1,200 bp, 900 bp, 700 bp, 500 bp, 300 bp, 100 bp); lanes 1-8, PCR products. C) The transcript levels of *TvTLP* in *T. vaporariorum* adults feeding on

transgenic tobacco for 5 days were determined by qPCR. D) Survival rate of *T. vaporariorum* adults on transgenic-*EGFP* and transgenic-*TvTLP* tobacco plants, with or without exposure to *B. bassiana* for 7 days. E) Survival rate of *T. vaporariorum* adults on transgenic-*EGFP* and transgenic-*TvTLP* tobacco plants, with or without exposure to *M. robertsii* for 7 days. F) The hatching success of *T. vaporariorum* on transgenic-*EGFP* and transgenic-*TvTLP* tobacco plants, with or without exposed to *B. bassiana*. G) The hatching success of *T. vaporariorum* on transgenic-*EGFP* and transgenic-*TvTLP* tobacco plants, with or without exposure to *M. robertsii*. H) *B. bassiana*-covered cadavers of *T. vaporariorum* nymph on transgenic-*EGFP* and transgenic-*TvTLP* tobacco plants. The white arrows indicate fungus-infected nymphs. I) *M. robertsii*-covered cadavers of *T. vaporariorum* nymph on transgenic-*EGFP* and transgenic-*TvTLP* tobacco plants. The white arrows indicate fungus-infected nymphs. Date are means ± SEM, $n = 3$ (C), $n = 10$ (D-G) biologically independent samples, ns, not significant, $^{**}P < 0.01$, $^{***}P < 0.001$, one-way ANOVA with Tukey's test was used for comparison.

A

Fungus	IC_{50} (95% FL)	Fungus	IC_{50} (95% FL)
B. bassiana	1.531 (1.348-1.742)	*M. robertsii*	2.043 (1.725-2.446)

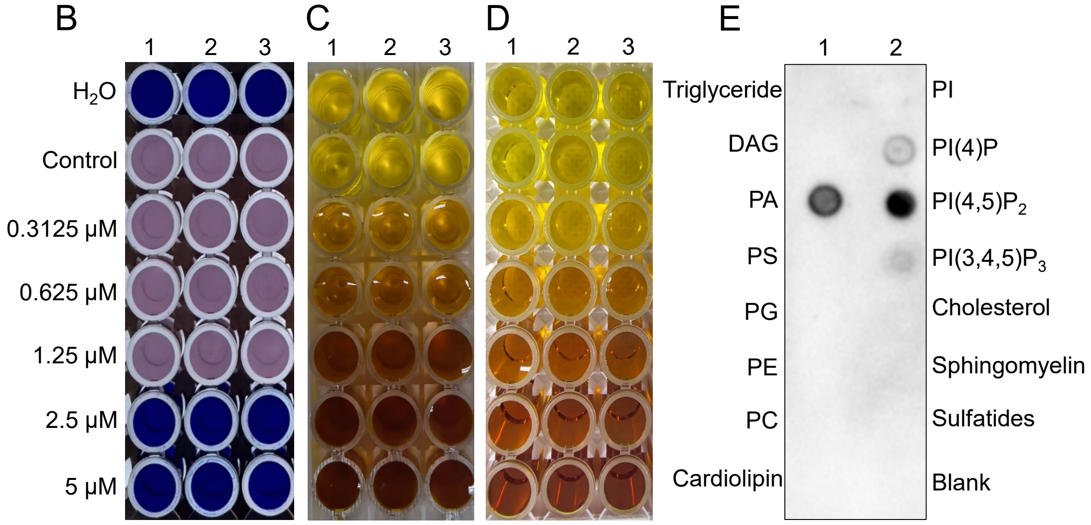

Figure S10. TvTLP has antifungal activity. A) The IC_{50} values of TvTLP for *B. bassiana* and *M. robertsii*. IC_{50} (95% FL), the concentration of TvTLP that kills 50% of *B. bassiana* and *M. robertsii* conidia with the associated 95% confidence limit. B) The fungal cell viability assay using resazurin. A change from blue to pink indicates metabolically active fungal conidia. The control water group without *B. bassiana* and *M. robertsii* conidia and TvTLP protein, the control group was added with *B. bassiana* conidia but without TvTLP protein. The numbers 1, 2, and 3 represent three repetitions. C-D) The respective β-1,3-glucanase activities of NtTLP and TvTLP. The color changes from yellow to brown, indicating an increase in β-1,3-glucanase activity. The numbers 1, 2, and 3 represent the three replications. E) Membrane Lipid Strip assay of TvTLP protein binding to membrane lipids. The intensity of the black dots correlates with the strength of the binding ability. DAG, diacylglycerol; PA, phosphatidic acid; PS, phosphatidylserine; PG, phosphatidylglycerol; PE, phosphatidylethanolamine; PC, Phosphatidylcholine; PI, phosphatidylinositol; PI(4)P, phosphatidylinositol 4-phosphate; PI(4,5)P2, phosphatidylinositol 4,5-bisphosphate; PI(3,4,5)P3, phosphatidylinositol 3,4,5-trisphosph.

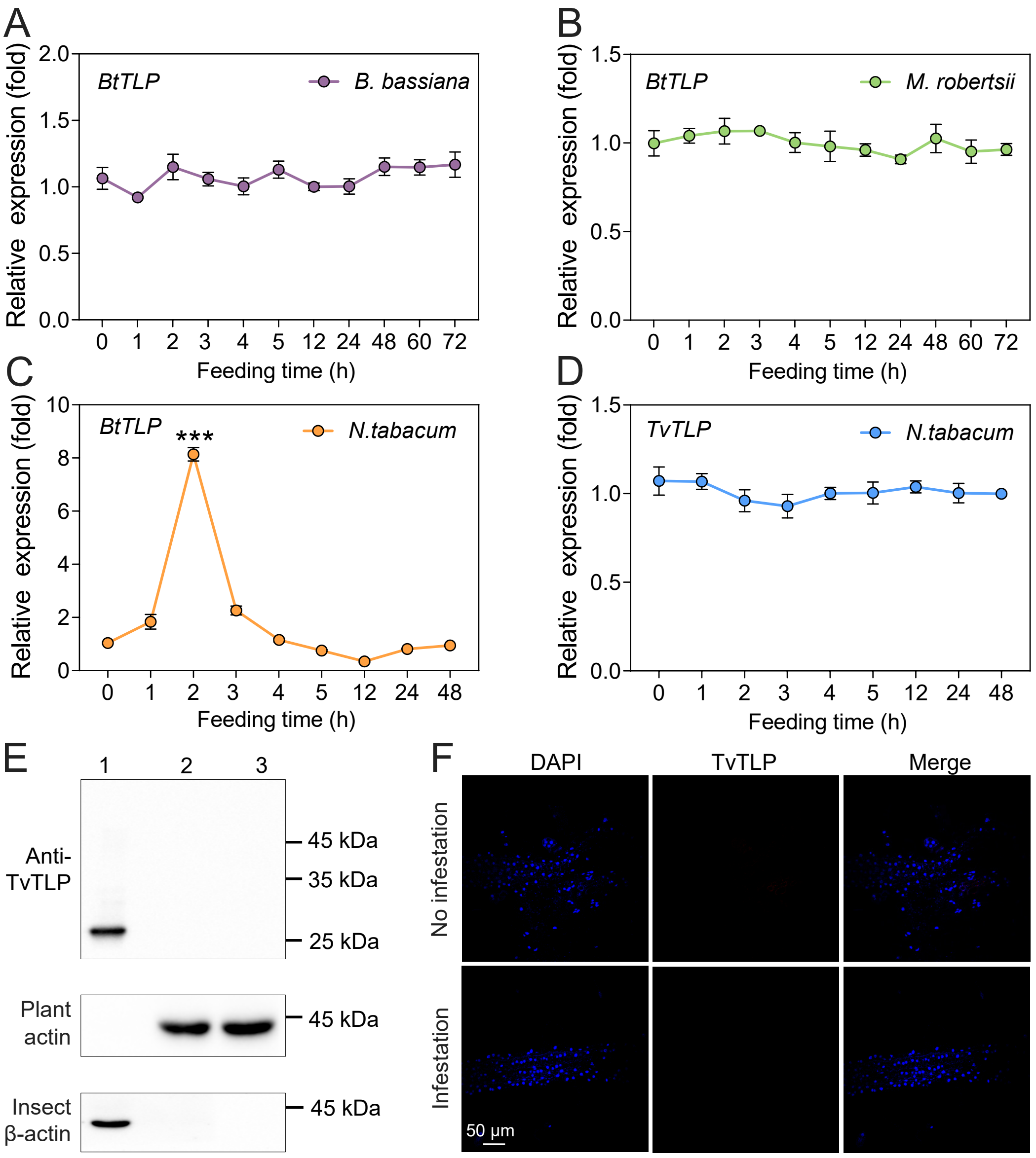

Figure S11. The transcript levels of *BtTLP* and *TvTLP* response to *B. bassiana*, *M. robertsii* and *N. tabacum*. A-B) The transcript levels of *BtTLP* after *B. tabaci* feeding on artificial diet with *B. bassiana* (A) and *M. robertsii* (B) spore suspension for 0 h, 1 h, 2 h, 3 h, 4 h, 5 h, 12 h, 24 h, 48 h, 60 h and 72 h were determined by qPCR. C) The transcript levels of *BtTLP* after *B. tabaci* had been feeding on tobacco plants for 1 h, 2 h, 3 h, 4 h, 5 h, 12 h, 24 h and 48 h as determined by qPCR. D) The transcript levels of *TvTLP* and after *T. vaporariorum* feeding on tobacco plants for 0 h, 1 h, 2 h, 3 h, 4 h, 5 h, 12 h, 24 h and 48 h were determined by qPCR. E) Western blot analysis of TvTLP

secretion. Lane 1, protein extracted from *T. vaporariorum* adults; lane 2, protein extracted from uninfested tobacco leaves; lane 3, protein extracted from *T. vaporariorum*-infested tobacco leaves. F) Immunofluorescence localization of TvTLP in *T. vaporariorum*-infested and uninfested tobacco leaves. DAPI is shown in blue. Date are means ± SEM, n = 3 (A-D) biologically independent samples, $^{***}P < 0.001$, one-way ANOVA with Tukey's test was used for comparison.

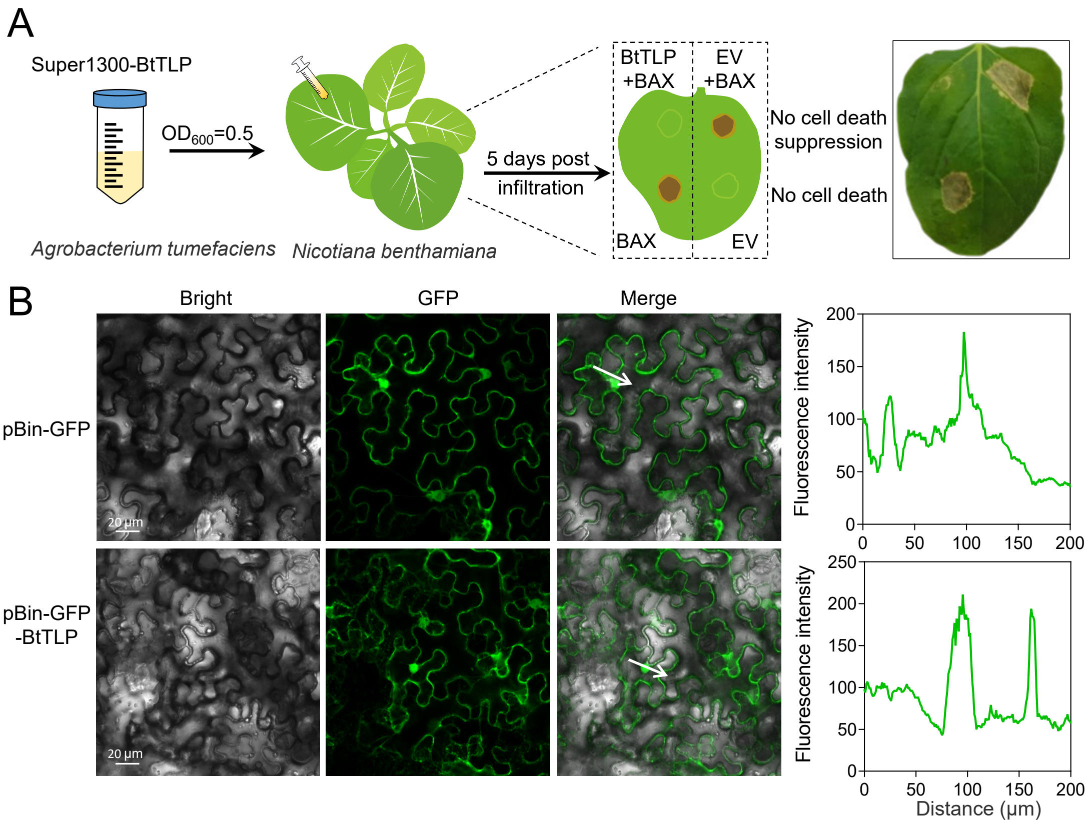

Figure S12. Transient expression of BtTLP in *N. benthamiana*. A) Agroinfiltration-mediated BtTLP suppresses the Bax-induced hypersensitive response in *N. benthamiana* after five days. B) Subcellular localization of BtTLP in *N. benthamiana*. The pBin-GFP and pBin-GFP-BtTLP fluorescence were detected with confocal microscopy 48 h after infiltration. White arrows represent fluorescence intensity profiles in GFP fluorescence images.

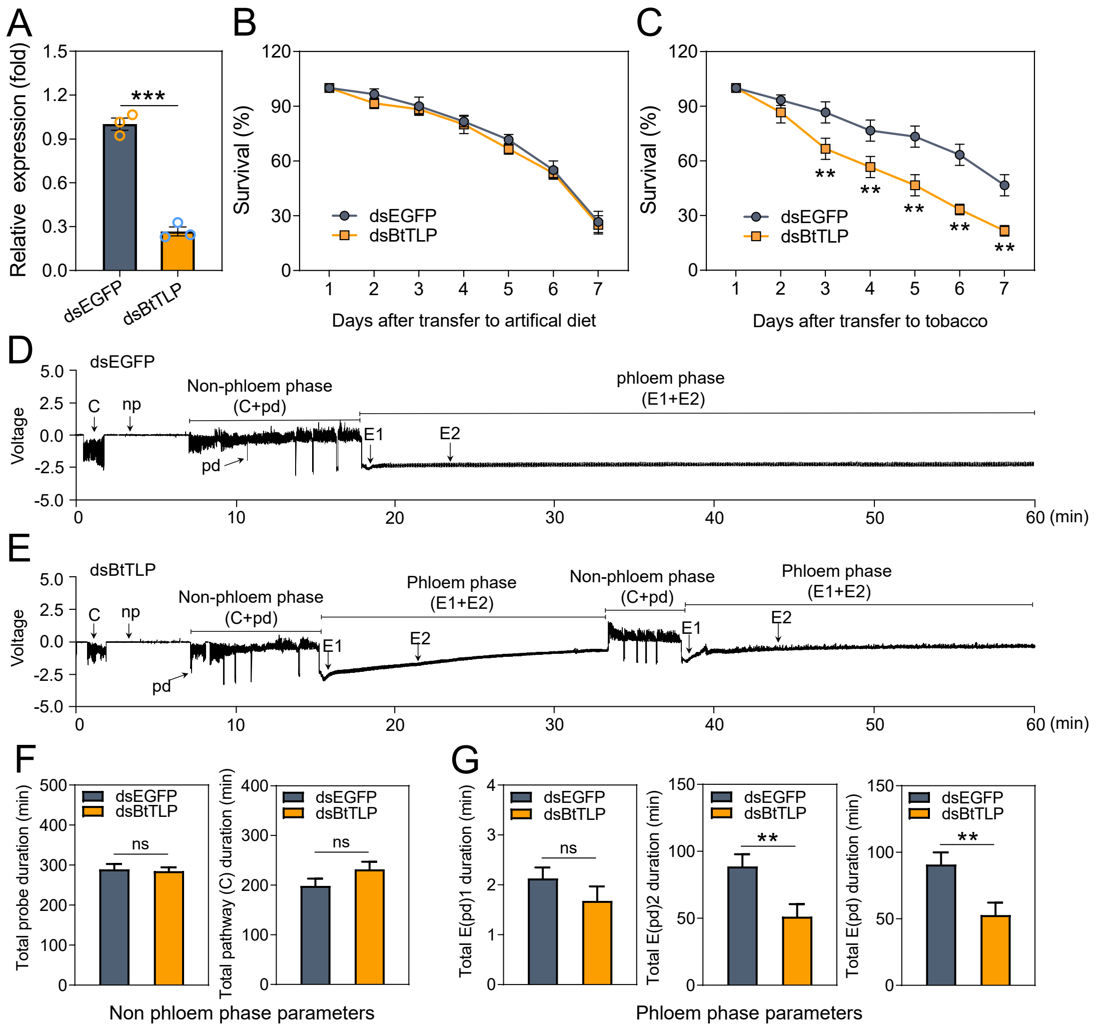

Figure S13. Effect of *BtTLP* silencing on *B. tabaci* performance. A) The transcript levels of *BtTLP* after RNAi treatment after 48 h of feeding were determined by qPCR. B-C) Survival rate of *B. tabaci* feeding on artificial diet (B) and tobacco plants (C) for 7 days. D-E) The EPG waveforms of dsEGFP treated (D) and dsBtTLP treated (E) *B. tabaci* for 1 h. F-G) Comparison of the non-phloem phase parameters (F) and phloem phase parameters (G) in dsEGFP and dsBtTLP fed *B. tabaci*. C, pathway phase; np, non-probing; pd, potential drop; E1, watery salivation; E2, passive phloem ingestion. Date are means ± SEM, n = 3 A-C), n = 16 (**F, G**) biologically independent samples, ns, not significant, $^{**}P < 0.01$, $^{***}P < 0.001$, one-way ANOVA with Tukey's test was used for comparison.

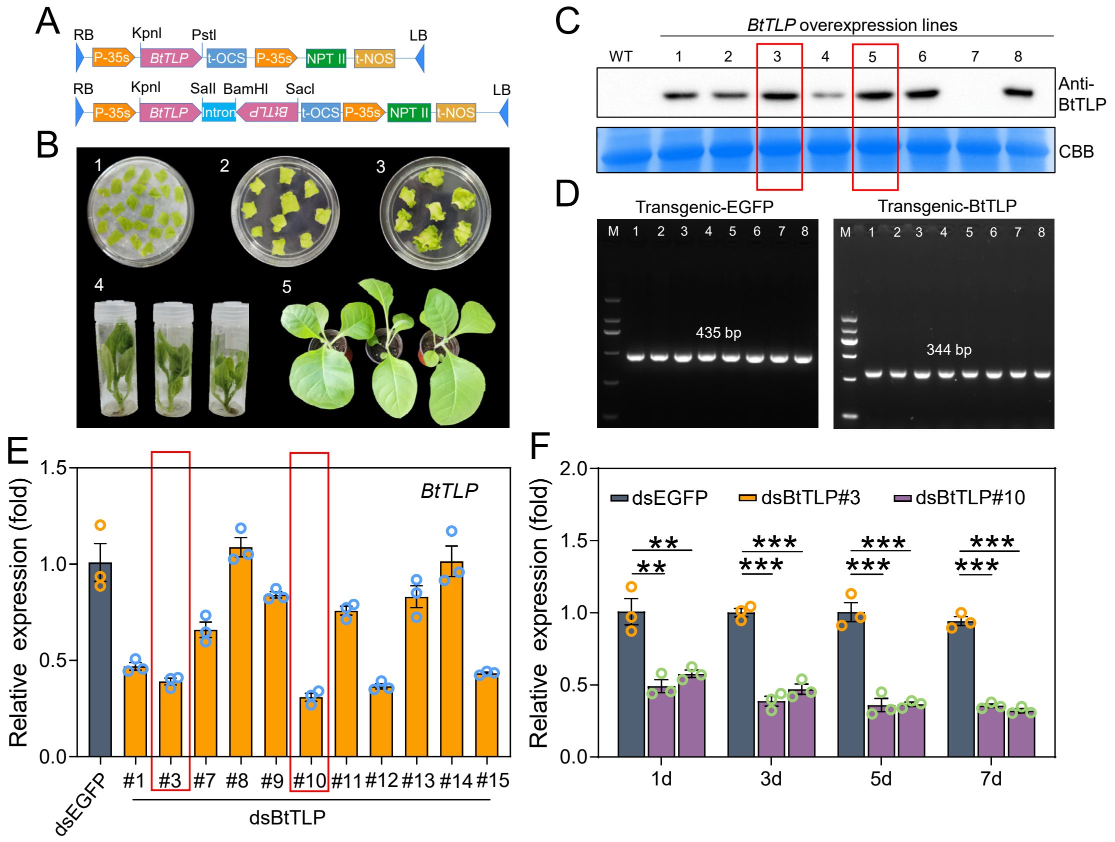

Figure S14. Construction and screening of *BtTLP* transgenic plants. A) The diagram of vector construction of RNAi and overexpression of *BtTLP* used for tobacco transformation. B) The transformation process of transgenic tobacco lines. C) Western blot detection of *BtTLP* in different transgenic tobacco lines. The *BtTLP* overexpression lines 3 and 5 were chosen for further study. Coomassie Brilliant Blue (CBB) staining showed the equal loading of each lane. D) PCR products amplified from transgenic-*EGFP* and transgenic-*BtTLP* tobacco leaves. M, marker (from top to bottom: 1,200 bp, 900 bp, 700 bp, 500 bp, 300 bp, 100 bp); lanes 1-8, PCR products. E) The transcript levels of *BtTLP* in *B. tabaci* adults after feeding on different dsBtTLP transgenic tobacco lines were determined by qPCR. Transgenic tobacco lines 3 and 10 with silenced *BtTLP* were chosen for further study. F) The transcript levels of *BtTLP* in *B. tabaci* adults feeding on dsBtTLP transgenic tobacco line 3 and line 10 for 1, 3, 5, and 7 days were determined by qPCR. Date are means ± SEM, $n = 3$ (E, F) biologically independent samples, $^{**}P < 0.01$, $^{***}P < 0.001$, one-way ANOVA with Tukey's test was used for comparison.

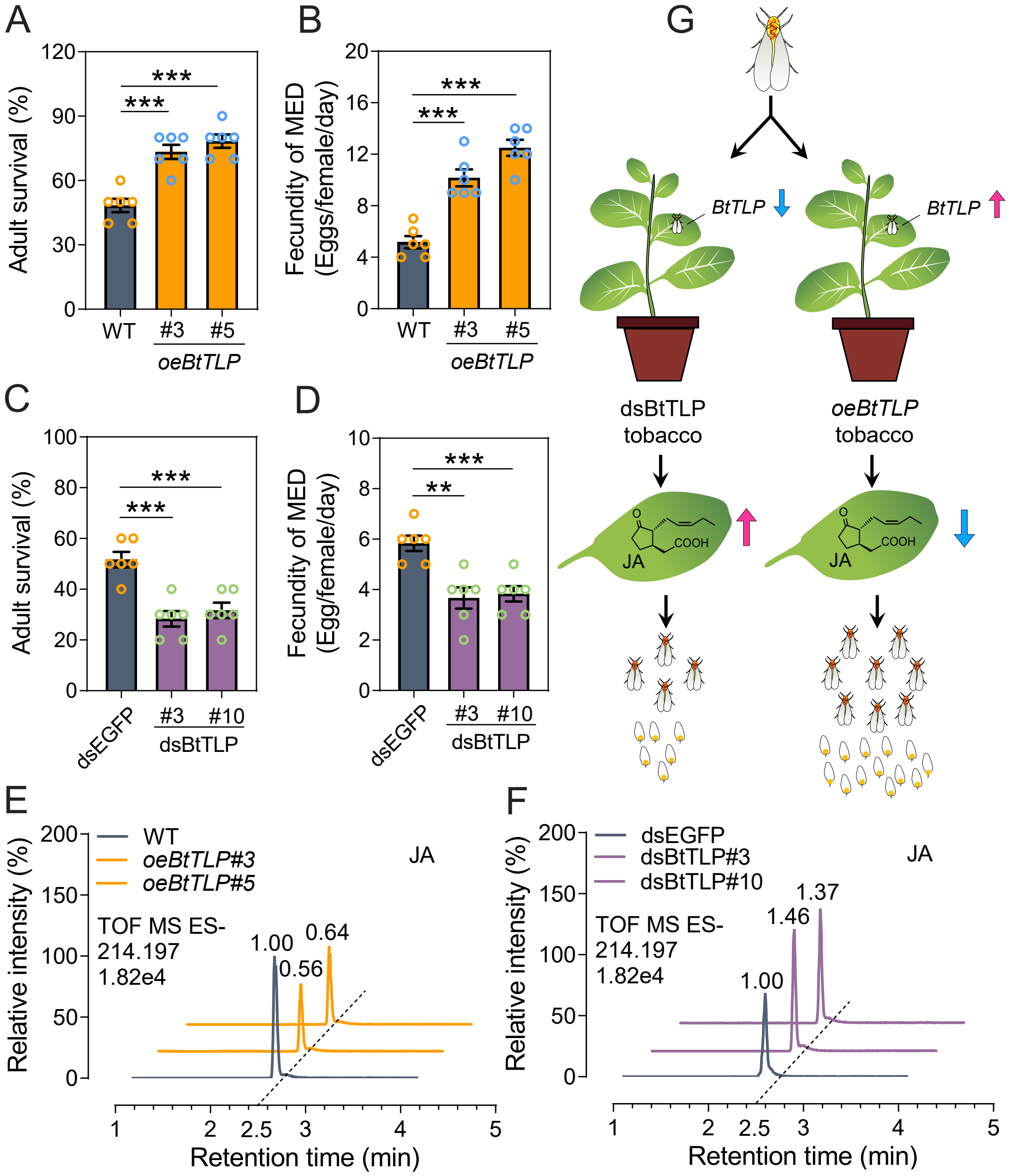

Figure S15. The performance of *B. tabaci* on transgenic tobacco plants. A-B) Survival rate (A) and fecundity (B) of *B. tabaci* MED on WT and two *BtTLP*-overexpressed (*oeBtTLP#3*, *oeBtTLP#5*) transgenic tobacco plants after 7 days of feeding. C-D Survival rate (C) and fecundity (D) of *B. tabaci* MED on WT and two *BtTLP*-silenced (dsBtTLP#3, dsBtTLP#10) transgenic tobacco plants after 7 days. E) The content of JA in WT and two *BtTLP*-overexpressed (*oeBtTLP#3*, *oeBtTLP#5*) transgenic tobacco plants. The numbers above each peak indicates the relative JA level calculated based

on the ratio of peak areas. F) The JA content in tobacco plants following infestation with *BtTLP*-silenced *B. tabaci* by two transgenic tobacco lines (dsBtTLP#3, dsBtTLP#10). The numbers above each peak indicates the relative JA level calculated by the ratio of peak areas. G) A proposed model of *B. tabaci* employing effector BtTLP to regulate plant JA and enhances the host adaptability. Date are means ± SEM, $n = 6$ (A-D) biologically independent samples, $*P < 0.05$, $**P < 0.01$, one-way ANOVA with Tukey's test was used for comparison.

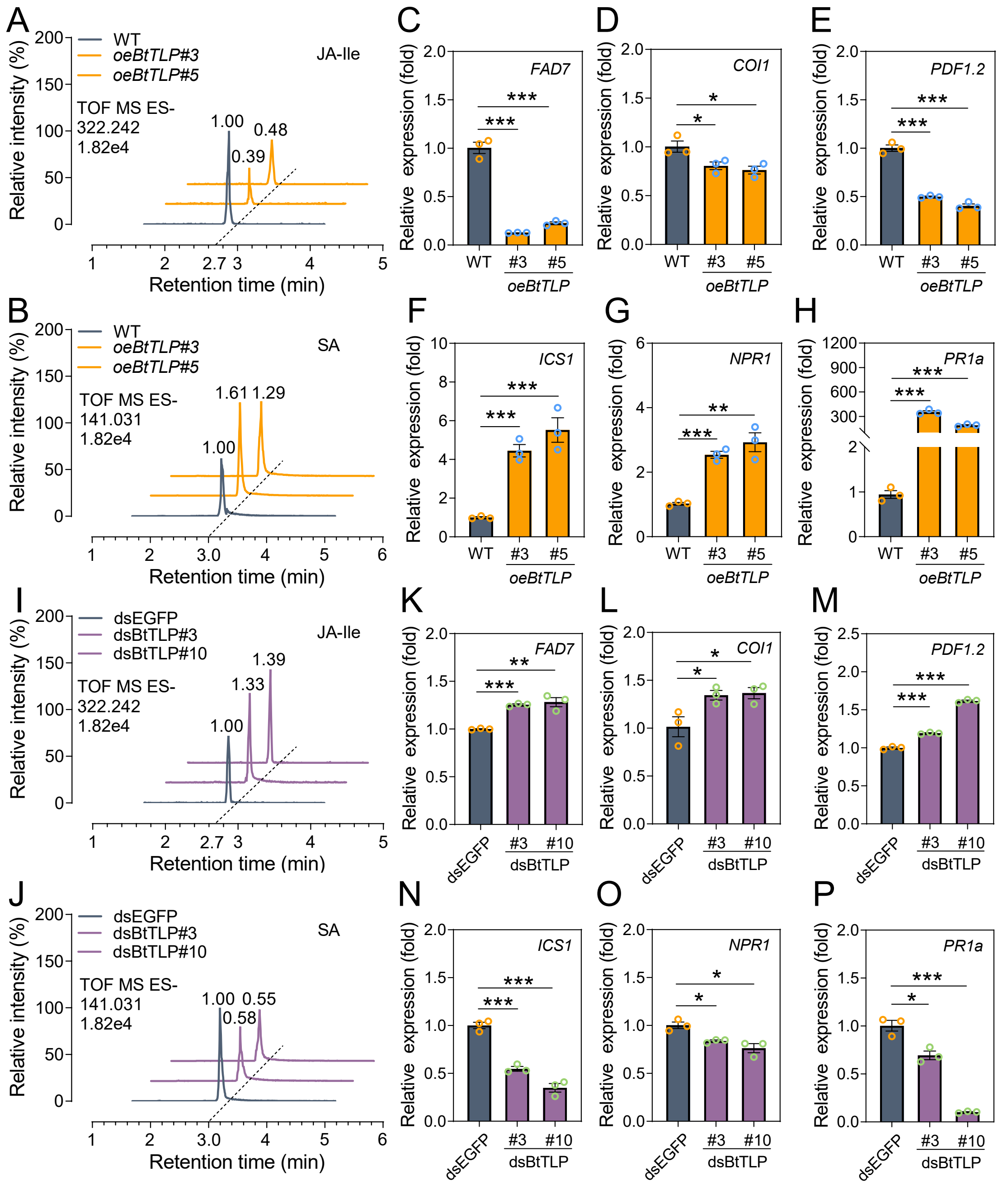

Figure S16. The content of JA-Ile and SA and the transcript level of JA-SA related marker genes in transgenic lines. A-B) The content of JA-Ile (A) and SA (B) in WT and two *BtTLP*-overexpressed (*oeBtTLP#3*, *oeBtTLP#5*) transgenic lines. The numbers above each peak indicates the relative JA level calculated based on the ratio of peak areas. C-E The transcript levels of JA related marker genes in WT and two *BtTLP*-overexpressed (*oeBtTLP#3*, *oeBtTLP#5*) transgenic lines were determined by qPCR. F-

H) The transcript levels of SA related marker genes in WT and two *BtTLP*-overexpressed (*oeBtTLP#3*, *oeBtTLP#5*) transgenic lines were determined by qPCR. I-J) The JA-Ile (I) and SA (J) content in tobacco plants following infestation with *BtTLP*-silenced *B. tabaci* by two transgenic tobacco lines (dsBtTLP#3, dsBtTLP#10). The numbers above each peak indicates the relative JA level calculated based on the ratio of peak areas. K-M) The transcript levels of JA related marker genes in tobacco plants following infestation with *BtTLP*-silenced *B. tabaci* by two transgenic tobacco lines (dsBtTLP#3, dsBtTLP#10) were determined by qPCR. N-P) The transcript levels of SA related marker genes in tobacco plants following infestation with *BtTLP*-silenced *B. tabaci* by two transgenic tobacco lines (dsBtTLP#3, dsBtTLP#10) were determined by qPCR. Date are means ± SEM, $n = 6$ (C-H, K-P) biologically independent samples, *$P < 0.05$, **$P < 0.01$, ***$P < 0.001$, one-way ANOVA with Tukey's test was used for comparison.

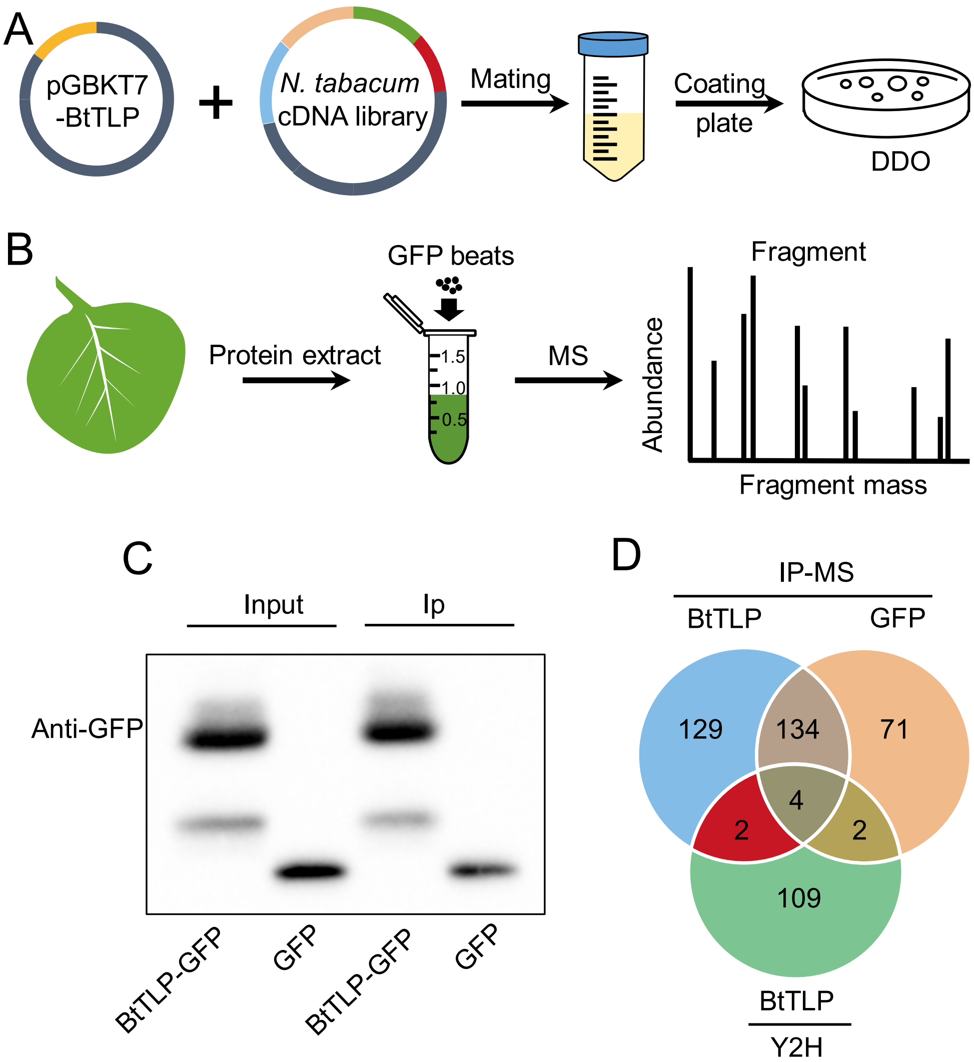

Figure S17. Identification of the potential host targets of BtTLP. A) The diagram of Y2H screening for BtTLP. B) The diagram of IP-MS for BtTLP. C) Western blot detection of BtTLP-GFP and GFP in input group and ip group. D) The Venn diagram illustrates that Y2H and IP-MS screened a substantial number of potential BtTLP interacting proteins.

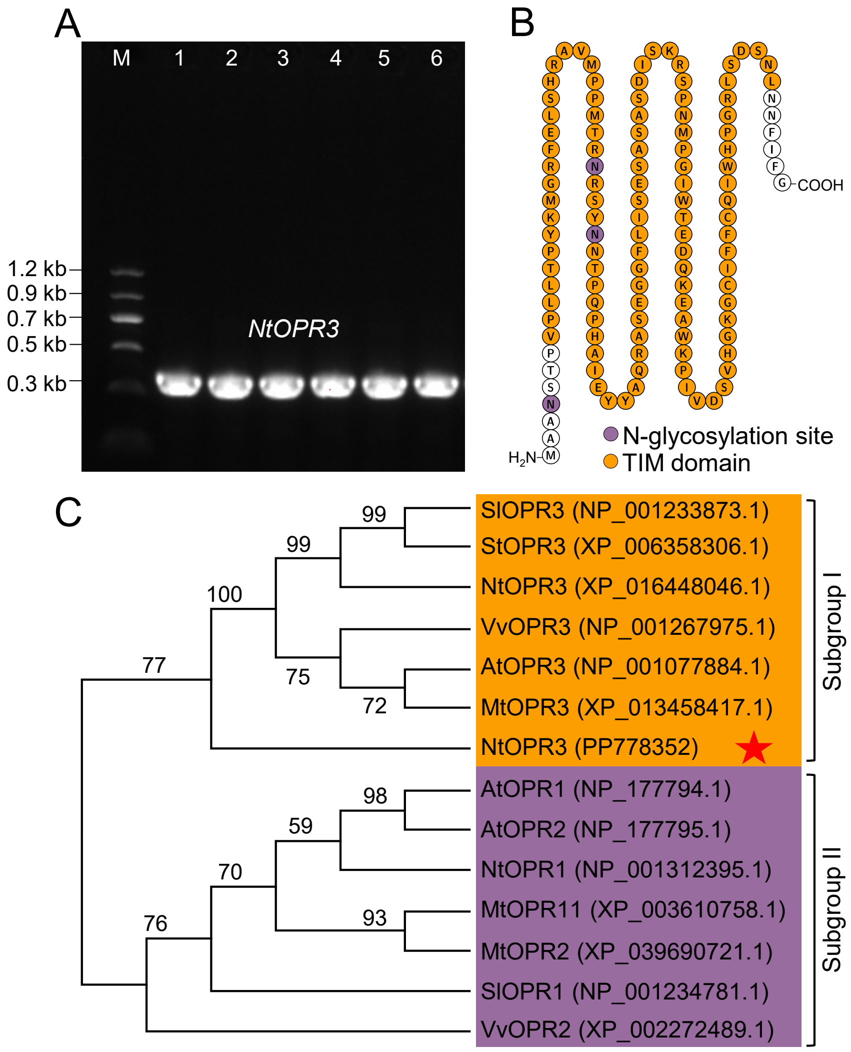

Figure S18. Cloning and characterization of *NtOPR3* in *N. tabacum*. A) *NtTLP* gene cloned from *N. tabacum*. Lanes 1-6, PCR products. B) The structure of NtOPR3 protein was generated by Protter. The N-glycosylation site and the TIM domain were labeled in purple and yellow. C) Phylogenetic analysis of OPR and it was drawn with the conserve domain of OPR. NtOPR3 is indicated by the red star.

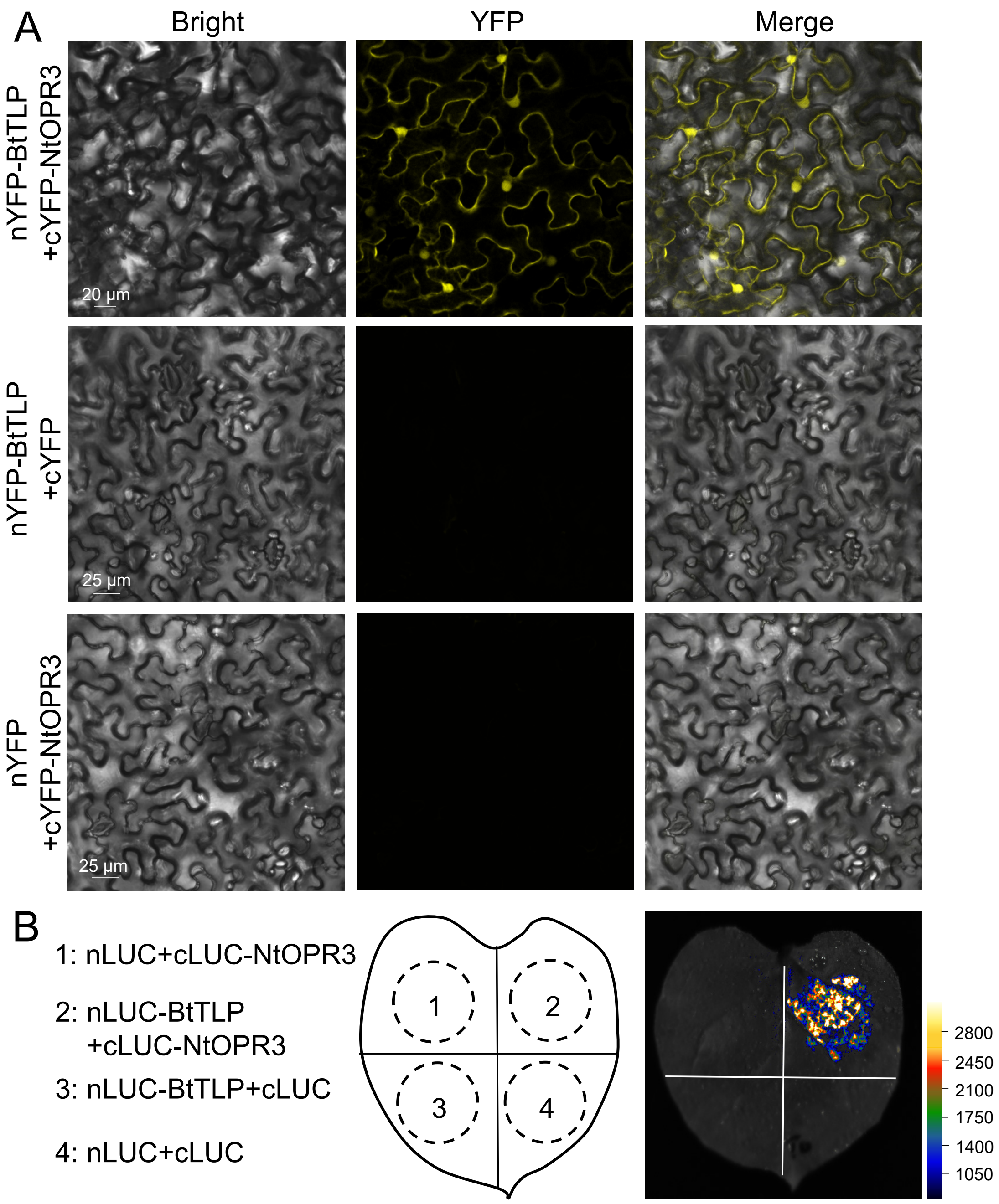

Figure S19. Analysis of the interaction between BtTLP and NtOPR3. A) BiFC assays showing the interaction between BtTLP and NtOPR3 in *N. benthamiana* leaves. B) Luciferase complementation (LUC) assays showing the interaction between BtTLP and NtOPR3.

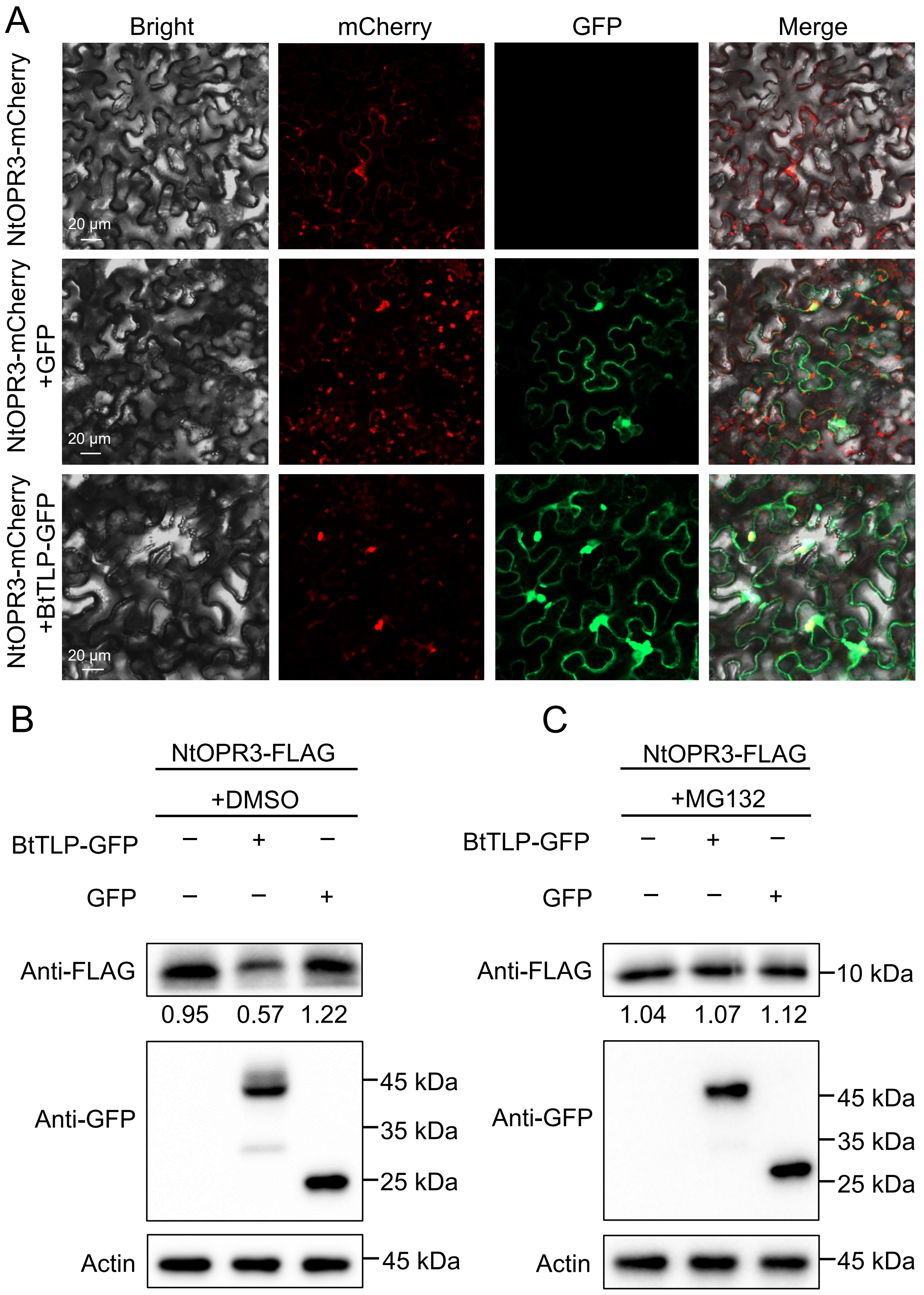

Figure S20. Analyses showing the effect of BtTLP on NtOPR3. A) Co-localization of BtTLP and NtOPR3. The co-localization of GFP and NtOPR3-mCherry was used as a control. B) Effects of BtTLP and GFP on NtOPR3 levels in *N. benthamiana*. C) Effects of the 26S proteasome inhibitor MG132 on NtOPR3 levels in *N. benthamiana*.

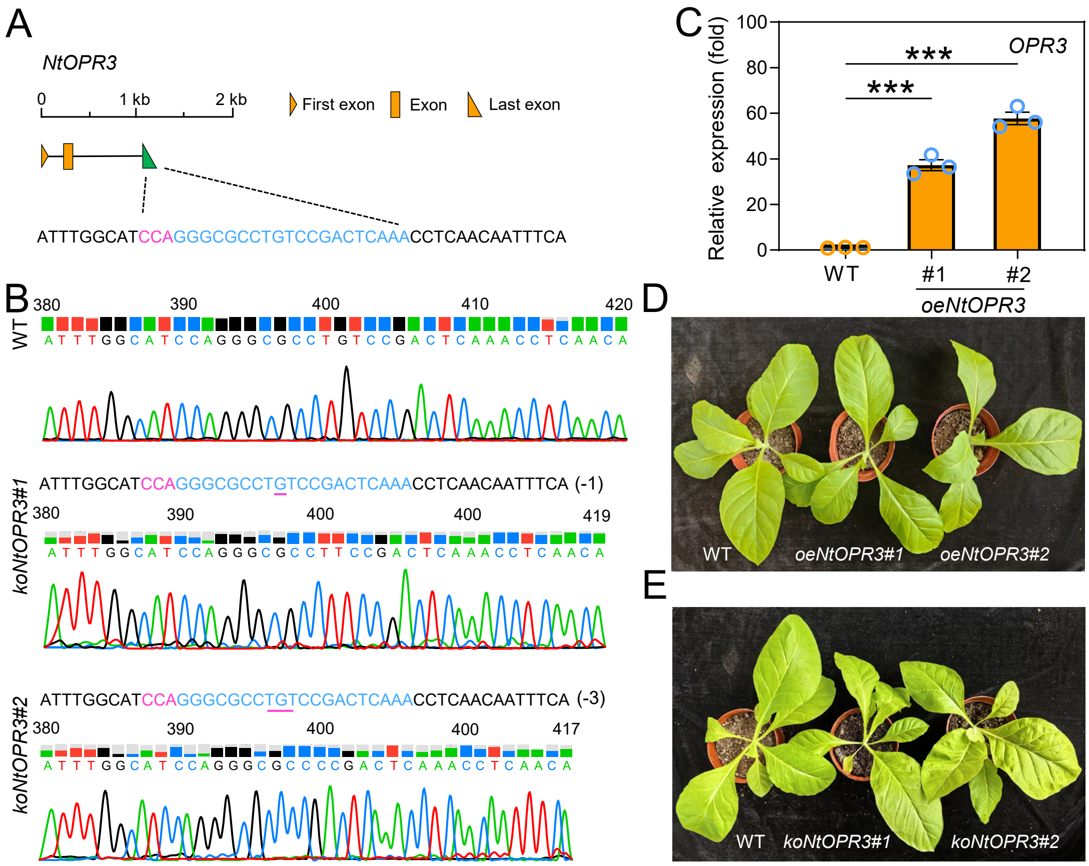

Figure S21. Analysis of the editing sequence and the transcript level of *NtOPR3* in transgenic tobacco plants. A) The designed sgRNA-targeted sites and sequences in exon 3 (green boxes) of *NtOPR3*. Exons are shown as orange boxes and green triangle, the spaces between two exons indicate the introns. The sgRNA of target gene is highlighted in blue, the PAM sequences are marked in pink. B) Sanger sequencing in WT and two independent mutations of *koNtOPR3* plants. The locations of CRISPR/Cas9-induced 1-bp deletion (G) of *koOPR3#1* and 3-bp deletion (TGT) of *koOPR3#2* within exon 3 of *NtOPR3* are marked by pink lines. C) The transcript levels of *NtOPR3* in WT and two *NtOPR3*-overexpressed transgenic tobacco plants were determined by qPCR. D) Phenotypes of WT and two *NtOPR3*-overexpressed transgenic tobacco plants. E) Phenotypes of WT and the two *NtOPR3* mutant transgenic tobacco plants. Date are means ± SEM, $n = 3$ (C) biologically independent samples, $^{***}P < 0.001$, one-way ANOVA with Tukey's test was used for comparison.

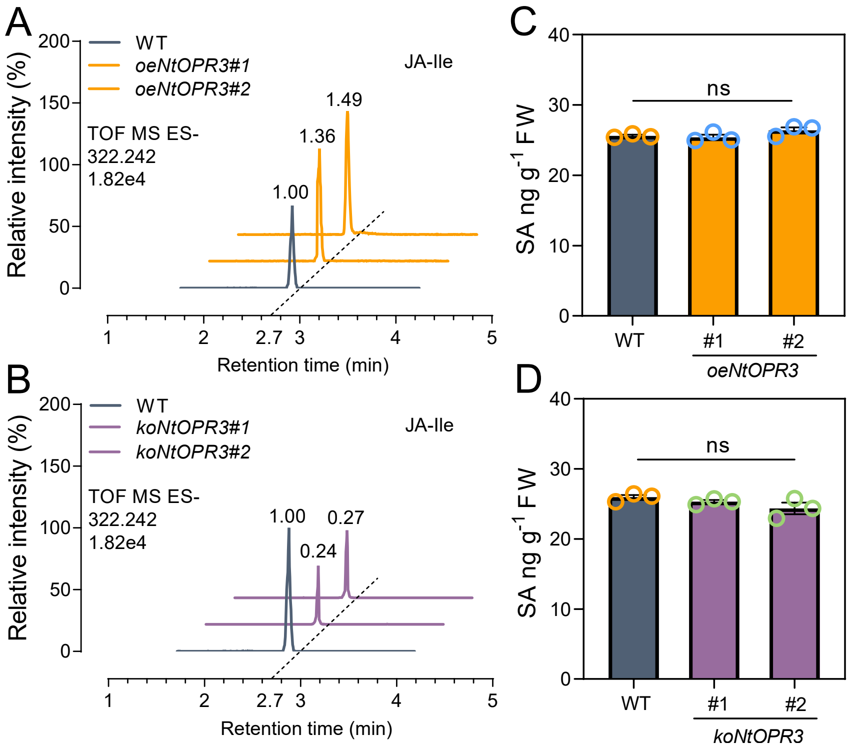

Figure S22. The content of JA-Ile and SA in transgenic tobacco lines. A-B) The content of JA-Ile in WT, two *NtOPR3*-overexpressed (*oeNtOPR3#1*, *oeNtOPR3#2*) transgenic tobacco lines (A) and two *NtOPR3* mutant (*koNtOPR3#1*, *koNtOPR3#2*) transgenic tobacco lines (B). The numbers above each peak indicates the relative JA-Ile level calculated by the ratio of peak areas. C-D) The content of SA in two *NtOPR3*-overexpressed (*oeNtOPR3#1*, *oeNtOPR3#2*) transgenic tobacco lines (C) and two *NtOPR3* mutant (*ktOPR3#1*, *koNtOPR3#2*) transgenic tobacco lines (D). Date are means ± SEM, n = 3 (A, B) biologically independent samples, ns, not significant, one-way ANOVA with Tukey's test was used for comparison.

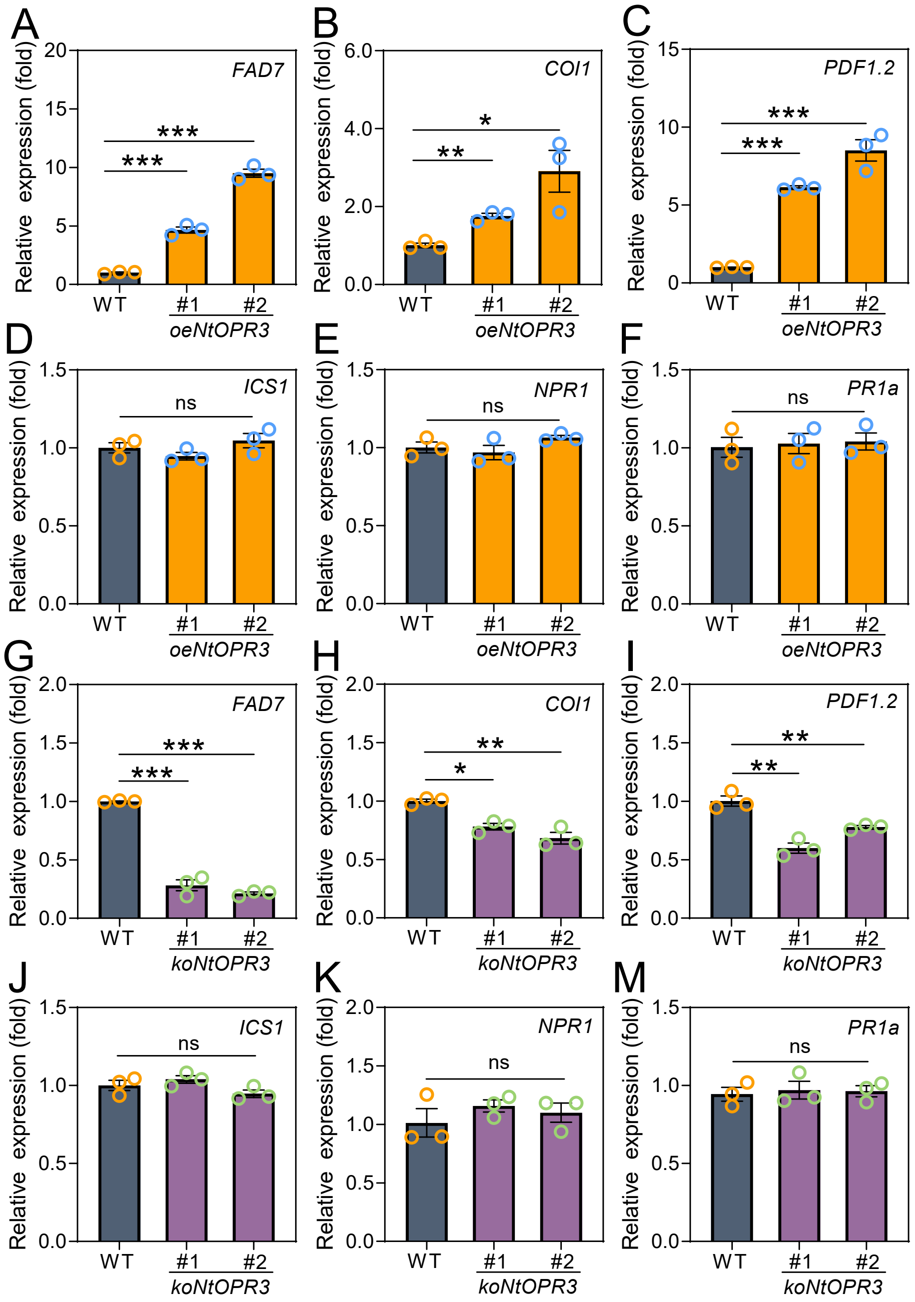

Figure S23. The transcript level of JA-SA related marker genes in transgenic lines. A-C) The transcript levels of JA related marker genes in two *NtOPR3*-overexpressed (*oeNtOPR3#1*, *oeNtOPR3#2*) transgenic tobacco lines were determined by qPCR. D-F) The transcript levels of SA related marker genes in two *NtOPR3*-overexpressed (*oeNtOPR3#1*, *oeNtOPR3#2*) transgenic tobacco lines were determined by qPCR. G-I) The transcript levels of JA related marker genes in two *NtOPR3* mutant (*koNtOPR3#1*,

koNtOPR3#2) transgenic tobacco lines were determined by qPCR. J-M) The transcript levels of SA related marker genes in two *NtOPR3* mutant (*koNtOPR3#1*, *koNtOPR3#2*) transgenic tobacco lines were determined by qPCR. Date are means ± SEM, $n = 3$ (A-M) biologically independent samples, ns, not significant, *$P < 0.05$, **$P < 0.01$, ***$P < 0.001$, one-way ANOVA with Tukey's test was used for comparison.

Table S1. Primers used in this study

Purpose	Gene name	Primer name	Primer sequences (5′-3′)	Tm (°C)	PCR product (bp)
Gene cloning	NtTLP	fNtTLP-F	ATGGCGCATGTGAAAGTACTC	57	990
		fNtTLP-R	TTAGAAGAGGTGACGCAATTGG		
	TvTLP	fTvTLP-F	CGGTGGCGAAAAATGACTTCAGCGT	65	792
		fTvTLP-R	CCTTATCCGATTCTGCCCGTGAGTT		
	BtTLP	fBtTLP-F	CTTCACTGTTGTTCGTGCATCTC	55	805
		fBtTLP-R	GAGGGCGACAGCCGTGATAGCT		
	NtOPR3	fNtOPDA-F	ATGGCAGCAAACTCCACCC	58	369
		fNtOPDA-R	CTAACCAAAAATGAAATTGTTGAGGT		
qPCR	NtTLP	qNtTLP-F	GAAAGTGACGAGCGTGGGAG	60	115
		qNtTLP-R	CGGTTTACAGATGTTGGGACTATT		
	TvTLP	qTvTLP-F	ATTTCAGATGTGAGACAGGAGATTG	60	118
		qTvTLP-R	TCGTAGAAATCTTGACCTCCGT		
	BtTLP	qBtTLP-F	ATGTTCATCAAGTTTGTGGTATCTC	60	89
		qBtTLP-R	CAGTTGTTCTTCACCGTTATCC		
	NtOPR3	qNtOPR3-F	CCTCTCCTTACTCCTTACAAAATGG	60	112
		qNtOPR3-R	CAATAGCATGTGGTTGTGGAGTATT		
	ICS1	qICS1-F	ATTCCTCTTCTAAAGTGGTCAGTGT	60	114
		qICS1-R	TCTTGGAGAGGAACCTGCGT		
	NPR1	qNPR1-F	ACTACAGATGTTGCTAAGAGAGGGG	60	122
		qNPR1-R	CATCAGCAAGTGCAAGATCTAGAAG		
	PR1a	qPR1a-F	TGCCTTCATTTCTTCTTGTCTCTAC	60	144
		qPR1a-R	CGTCCCAGGTCAAAGGTTCTAC		
	FAD7	qFAD7-F	AGTTATGTTGTGAGGGATGTTGCTA	60	127
		qFAD7-R	GAACAAAGAGAGCCCAAAACATAGT		
	COI1	qCOI1-F	ATGTTACACCTTGGGTAGTGGAAA	60	129
		qCOI1-R	CCTGAAGCACTTTACCCCGA		
	PDF1.2	qPDF1.2-F	CTACCGAGATGGGACCAATGA	60	124

		qPDF1.2-R	GAAAATCCTTCGGTCAGACAAAC		
	GAPDH	qGAPDH-F	GACTCTGACGGACTTCTCACCAA	60	137
		qGAPDH-R	GACTCTGACGGACTTCTCACCAA		
	EF1α	qEF1α-F	TAGCCTTGTGCCAATTTCCG	60	110
		qEF1α-R	CCTTCAGCATTACCGTCC		
Genome fragment	TvTLP-upstream	upTvTLP-1F	CTGGAAGTTCCAGATTGCA	64	5245
		upTvTLP-1R	CGAATGCGAAGGTGTTCACCAAAC		
		upTvTLP-2F	TTCTGGACAAATTTATAGGATCA	61	5005
		upTvTLP-2R	GTCCGTTTCCCGAAATATTCC		
		upTvTLP-3F	CACTCGCAAATGCTTACGGG	62	3013
		upTvTLP-3R	ATCAAATGAATCGTCAAAAACTA		
	TvTLP-downstream	upTvTLP-4F	CAAGGTCTTCAGAGAATAC	55	5479
		upTvTLP-4R	CTATGATGATCTTAGAGGTAC		
		downTvTLP-1F	TAGCCCTTAAAGGAGTACACT	64	5059
		downTvTLP-1R	AACAGTCGCTAGGTGTCACGC		
		downTvTLP-2F	TTGAAAAGTTCGTTGTTTCCG	59	5210
		downTvTLP-2R	GGGAAAAAGCATTGAATCAG		
		downTvTLP-3F	TGACTAGAACGCTTCCGACT	56	4130
		downTvTLP-3R	ATAATTGTAATCATAGTGA		
		downTvTLP-4F	GGAGATTCCCACCACGCACAT	66	3826
		downTvTLP-4R	GCTCGGAATGCCAATTTTGGATA		
	BtTLP-upstream	upBtTLP-1F	GTACGAGAGCTCTTCATAGTGACT	65	4011
		upBtTLP-1R	TCCCGCTGGGGGAAAATGAA		
		upBtTLP-2F	TTTCGTTTTTCAGTTCCCAATTTCT	65	3575
		upBtTLP-2R	CAACTGAAACTAGACCCTGGGTAGA		
		upBtTLP-3F	CCCTGATTTTGTGTTTTGCAAT	66	4320
		upBtTLP-3R	CCCCCGGGGCTTTCTCGG		
	BtTLP-downstream	downBtTLP-1F	TAACTACGTCGTCACCTTCTGC	64	4043
		downBtTLP-1R	ATTCACTTTCTTCTCGGGAAACT		
		downBtTLP-2F	ATGACGAGGTGTGTTTCTTAATCG	67	3831

		downBtTLP-2R	GGAGTCGGGTGCCGATACCGA		
		downBtTLP-3F	CATGATAATCGATTCTTCGCC	61	3650
		downBtTLP-3R	GTGTCTACAAGTTCGGAAAGT		
		downBtTLP-4F	TTCAGTACTTCCGGACCGCGCTT	59	4270
		downBtTLP-4R	GGAATCAAATTTTCCAAATTCT		
		downBtTLP-5F	CTCGCCCGCATTATTACACAATT	58	4343
		downBtTLP-5R	GTTAATACATACTAAAAGTTC		
		downBtTLP-6F	CTTTTCTCTCTCATCCTTGCCG	63	4180
		downBtTLP-6R	ATTGCTACTAAGTAGGCATGCAA		
		downBtTLP-7F	TTTCCCACTGCAGTGTAGCCG	66	4102
		downBtTLP-7R	CATCTGCAAACAAAAGGAGAGGT		
dsRNA synthesis[1)]	TvTLP	dsTvTLP-F	TAATACGACTCACTATAGGGAGA<br>AGCGTCCAGACGACCCAGT	60	293
		dsTvTLP-R	TAATACGACTCACTATAGGGAGA<br>CTCCTTGCTGTGAGCAGCC		
	BtTLP	dsBtTLP-F	TAATACGACTCACTATAGGGAGA<br>TGTGGCCGGCGACTCAGA	60	344
		dsBtTLP-R	TAATACGACTCACTATAGGGAGA<br>GATGTGCCGGCACTTGGG		
	EGFP	dsEGFP-F	TAATACGACTCACTATAGGGAGA<br>CCACAAGTTCAGCGTGTCCG	60	435
		dsEGFP-R	TAATACGACTCACTATAGGGAGA<br>AAGTTCACCTTGATGCCGTTC		
Vector construction[2)]	BtTLP	pBin-GFP-BtTLP-F	GACGAGCTGTACAAGGGTACC<br>GTGCGGATAACGGTGAAGAACA	60	663
		pBin-GFP-BtTLP-R	TCATCTAGAGGATCCGTCGAC<br>TCAAGGGCAGAAGGTGACGA		
	BtTLP	BD-BtTLP-F	ATGGCCATGGAGGCCGAATTC<br>GTGCGGATAACGGTGAAGAACA	60	663
		BD-BtTLP-R	ATGCGGCCGCTGCAGGTCGAC		

		TCAAGGGCAGAAGGTGACGA		
NtOPR3	AD-NtOPR3-F	GCCATGGAGGCCAGTGAATTC<br>ATGGCAGCAAACTCCACCC	60	369
	AD-NtOPR3-R	CAGCTCGAGCTCGATGGATCC<br>CTAACCAAAAATGAAATTGTTGAGGT		
NtOPR3	Super1300-Flag-NtOPR3-F	CTGCAGGGGCCCGGGGTCGAC<br>ATGGCAGCAAACTCCACCC	60	366
	Super1300-Flag-NtOPR3-R	GTCTTTGTAGTCCATGGTACC<br>ACCAAAAATGAAATTGTTGAGGTTT		
BtTLP	NYFP-BtTLP-F	TCCATCGATAGTACTGTCGAC<br>ATGGTGCGGATAACGGTGAA	60	663
	NYFP-BtTLP-R	CTCCATCCCGGGAGCGGTACC<br>AGGGCAGAAGGTGACGACG		
NtOPR3	CYFP-NtOPR3-F	TCCATCGATAGTACTGTCGAC<br>ATGGCAGCAAACTCCACCCC	60	366
	CYFP-NtOPR3-R	GTACATCCCGGGAGCGGTACC<br>ACCAAAAATGAAATTGTTGAG		
BtTLP	Nluc-BtTLP-F	ACGGGGGACGAGCTCGGTACCATGG<br>ATTACAAGGATGACGACGATAAG<br>GTGCGGATAACGGTGAAGAACA	60	660
	Nluc-BtTLP-R	CGCGTACGAGATCTGGTCGAC<br>AGGGCAGAAGGTGACGACG		
NtOPR3	Cluc-NtOPR3-F	TACGCGTCCCGGGGCGGTACCATG<br>GCAGCAAACTCCACCCCAGT	60	363
	Cluc-NtOPR3-R	ACGAAAGCTCTGCAGGTCGACTCAAG<br>CGTAATCTGGAACATCGTATGGGTA<br>ACCAAAAATGAAATTGTTGA		
NtOPR3	Mcherry-NtOPR3-F	CTGCAGGGGCCCGGGGTCGAC<br>ATGGCAGCAAACTCCACCC	60	366
	Mcherry-NtOPR3-R	GCCCTTGCTCACCATGGTACC		

			ACCAAAAATGAAATTGTTGAGGTTT		
Transgenic plants[3)]	oeNtTLP	oeNtTLP-F	CATTTACGAACGATAGGTACC	60	990
			ATGGCGCATGTGAAAGTACTCTG		
		oeNtTLP-R	ACACATTGGCAAAGACTGCAG		
			TTAGAAGAGGTGACGCAATTGGC		
	oeBtTLP	oeBtTLP-F	CATTTACGAACGATAGGTACC	60	726
			ATGTTCATCAAGTTTGTGGTATCTCTC		
		oeBtTLP-R	ACACATTGGCAAAGACTGCAG		
			TCAAGGGCAGAAGGTGACGA		
	oeNtOPR3	oeNtOPR3-F	CATTTACGAACGATAGGTACC	60	369
			ATGGCAGCAAACTCCACCC		
		oeNtOPR3-R	ACACATTGGCAAAGACTGCAG		
			CTAACCAAAAATGAAATTGTTGAGGT		
	CRISPR-NtTLP[4)]	CRISPR-NtTLP-F	GATGGTCTCATGATTG	72	Exon 2
			**CTCGACGGTTCCGGCGGAA**		
			CAATCACTACTTCGACTCTAG		
		CRISPR-NtTLP-R	TGGGGTCTCTAAAC		
			**TTCCGCCGGAACCGTCGAG**		
			CAATCACTACTTCGACTCTAG		
	CRISPR-NtOPR3[5)]	CRISPR-NtOPR3-F	GATGGTCTCATGATTG	72	Exon 3
			**GGCGCCTGTCCGACTCAAA**		
			CAATCACTACTTCGACTCTAG		
		CRISPR-NtOPR3-R	TGGGGTCTCTAAAC		
			**TTTGAGTCGGACAGGCGCC**		
			CAATCACTACTTCGACTCTAG		
	dsTvTLP	Sense-dsBtTvTLP-F	ACGGGGGACAAGCTTGGTACC	60	293
			AGCGTCCAGACGACCCAGTG		
		Sense-dsTvTLP-R	GCGGTATATACGTACGTCGAC		
			CTCCTTGCTGTGAGCAGCCA		
		Anti-sense-dsTvTLP-F	GGCGTACGTGTGCAGGGATCC	60	293

		CTCCTTGCTGTGAGCAGCCA		
	Anti-sense-dsTvTLP-R	AATTCGAGCTGGTCAGAGCTC AGCGTCCAGACGACCCAGTG		
dsBtTLP	Sense-dsBtTLP-F	ACGGGGGACAAGCTTGGTACC TGTGGCCGGCGACTCAGA	60	344
	Sense-dsBtTLP-R	GCGGTATATACGTACGTCGAC GATGTGCCGGCACTTGGG		
	Anti-sense-dsBtTLP-F	GGCGTACGTGTGCAGGGATCC GATGTGCCGGCACTTGGG	60	344
	Anti-sense-dsBtTLP-R	AATTCGAGCTGGTCAGAGCTC TGTGGCCGGCGACTCACA		

[1)] The T7 promoter sequence is underlined. Their lengths were not calculated in the length of final PCR product.

[2), 3)] The adaptors are underlined. Their lengths were not calculated in the length of final PCR product.

[4), 5)] The sgRNA target sequences of the *NtTLP* and *NtOPR3* are underlined and in bold.

Table S2. The TLP BLAST hits in seven Aleyrodidae species (excel file).

Table S3. Five pairs of primers to clone *TvTLP* pseudogene (excel file).

Table S4. The potential target proteins of BtTLP (excel file).

Text S1. Sequences with GenBank accession nos. PP778349 to PP778352.
